# Epstein-Barr virus-associated tumors: commonalities in pathogenesis and the tumor immune microenvironment

**DOI:** 10.3389/fimmu.2026.1791347

**Published:** 2026-04-24

**Authors:** Xiyi Wang, Dongmei Hu, Renqin Li, Wenjun Liao, Chao Li, Qiuhan Liu, Jiayao Li, Qiaohua Li, Wenpei Guo, Liyang Wang, Shichuan Zhang, Yue Zhao

**Affiliations:** 1 Department of Radiation Oncology, Sichuan Cancer Hospital & Institute, School of Medicine, University of Electronic Science and Technology of China, Chengdu, Sichuan, China; 2 Department of Clinical Oncology, Radiation Oncology Key Laboratory of Sichuan Province, Sichuan Clinical Research Center for Cancer, Sichuan Cancer Hospital & Institute, Sichuan Cancer Center, Affiliated Cancer Hospital of University of Electronic Science and Technology of China, Chengdu, Sichuan, China; 3 Department of Radiation Oncology, Radiation Oncology Key Laboratory of Sichuan Province, Sichuan Clinical Research Center for Cancer, Sichuan Cancer Hospital & Institute, Sichuan Cancer Center, Affiliated Cancer Hospital of University of Electronic Science and Technology of China, Chengdu, Sichuan, China; 4 Department of Head and Neck Surgery, Radiation Oncology Key Laboratory of Sichuan Province, Sichuan Clinical Research Center for Cancer, Sichuan Cancer Hospital & Institute, Sichuan Cancer Center, Affiliated Cancer Hospital of University of Electronic Science and Technology of China, Chengdu, Sichuan, China; 5 School of Basic Medical Sciences, Chengdu University of Traditional Chinese Medicine, Chengdu, Sichuan, China; 6 School of Clinical Medicine, Southwest Medical University, Luzhou, Sichuan, China

**Keywords:** Epstein-Barr virus, oncogenesis, PD-L1, tumor microenvironment, tumor vaccine

## Abstract

Epstein-Barr virus (EBV) is associated with a wide spectrum of lymphoid and epithelial malignancies. This review provides a comprehensive pan-tumor perspective on the convergent oncogenic mechanisms, immune microenvironment characteristics, and shared therapeutic vulnerabilities across EBV-related cancers. We systematically summarize EBV infection and latency patterns, highlighting key shared signaling networks (e.g., NF-κB, PI3K/AKT) alongside nuanced immune evasion strategies, such as HLA modulation and steric immune blockade. By bridging these molecular insights with clinical translation, we evaluate emerging therapeutic paradigms, including immune checkpoint inhibitors, EBV-specific T-cell therapies, neutralizing antibodies, and mRNA vaccines. Ultimately, this synthesis underscores the potential of exploiting shared viral vulnerabilities to develop pan-tumor targeted therapies, offering a strategic roadmap for future clinical development.

## 1 Introduction

Epstein-Barr virus (EBV), also designated human herpesvirus 4 (HHV-4), is a γ- herpesvirus and the first identified human oncovirus. First identified in Burkitt lymphoma cells six decades ago, EBV is now established as a group I carcinogen, implicated in the pathogenesis of approximately 1.5% of global cancer burden. EBV-associated malignancies originate from distinct cellular lineages—spanning epithelial, T-cell, B-cell, and NK-cell origins—and include lymphoepithelial carcinoma (LEC), a significant subset of gastric carcinomas (EBVaGC), endemic Burkitt lymphoma (BL), a proportion of Hodgkin lymphomas (cHL), natural killer/T-cell lymphomas (NKTCL), and EBV-positive diffuse large B-cell lymphomas (DLBCL), among others (1, 2). These tumours exhibit marked geographic and ethnic disparities; for example, NPC incidence peaks in East and Southeast Asia (25–50 per 100, 000), while EBV-driven lymphomas predominate in Africa and Latin America (3, 4). Despite their clinicopathological heterogeneity, their shared viral etiology implies common oncogenic principles.

EBV is an enveloped icosahedral virion with a ~172 kbp double-stranded DNA genome encoding approximately 100 proteins (5). Its entry mechanisms differ between B cells and epithelial cells. In B cells, gp350/220 binds CD21, followed by gp42–HLA class II interaction and gH/gL–gB-mediated fusion. Epithelial infection occurs via integrin engagement by gHgL, cell-to-cell transfer, or lateral viral spread (6–8). It is estimated that more than 95% of adults worldwide carry asymptomatic, lifelong EBV infection. A defining feature of EBV pathogenesis is its ability to establish three distinct latency programs in a cell-dependent manner (**Table 1**). Latency refers to a quiescent state in which EBV maintains its genome without virion production, while expressing a restricted set of viral genes to regulate host cell survival, proliferation, and immune evasion. Type I latency expresses only EBNA1 to sustain viral episomes. Type II latency expresses EBNA1 plus LMP1 and LMP2, which mimic receptor signaling to drive oncogenic pathways. Type III latency expresses the full panel of latent genes, causing potent cell transformation, typically in immunocompromised settings. Viral genomes persist as episomes in memory B cells, while epithelial malignancies often emerge when latent infection is stabilized by premalignant mutations (9–11).

**Table 1:** EBV latency programs and viral gene expression in associated tumors.

Tumor type	Latency	EBV genes expressed
Nasopharyngeal carcinoma	I/II	EBNA1,LMP1,LMP2A,EBERs,miR-BARTs
Intrahepatic cholangiocarcinoma	I	EBERs,EBNA1(LMP1/2 absent or variable)
Gastric cancer	I/II	EBNA1,EBERs,miR-BARTs,LMP2A(LMP1 usually negative)
Lymphoepithelial carcinoma	II	EBNA1,LMP1,LMP2A,EBERs
Diffuse large B cell lymphoma	III	EBNA1,EBNA-LP,EBERs,LMP1,LMP2A/B(EBNA3 family expressed in a subset of cases)
NK/T cell lymphoma	I/II	EBNA1,LMP1,LMP2A,EBERs(EBNA2 usually negative)
Burkitt’s lymphoma	I	EBNA1,EBERs,miR-BARTs(LMP1,LMP2 usually negative)
Hodgkin’s lymphoma	II	EBNA1,LMP1,LMP2A,EBERs(EBNA2 usually negative)

A core mechanism of EBV oncogenesis is the constitutive activation of key proliferative and pro-survival pathways, including NF-κB, PI3K/AKT, and JAK/STAT, driven by latent proteins and non-coding RNAs. EBV also profoundly subverts host immunity by impairing antigen presentation, upregulating immune checkpoints such as PD-L1, and remodeling the tumor microenvironment (TME) into an “immune-hot but functionally suppressed” state (12, 13). Although EBV elicits robust T-cell responses during acute infection, it evades elimination in tumors through mechanisms including EBNA1’s Gly–Ala repeat–mediated avoidance of proteasomal degradation (14, 15) and LMP1-driven NF-κB activation (16, 17).

Notably, the virus blurs the boundary between latency and lytic replication. Reactivation, triggered by the immediate-early transactivators BZLF1 and BRLF1, can be accelerated by diverse stimuli, including phorbol esters, BCR engagement, HDAC inhibitors, viral kinases, sodium butyrate, hypoxia, or TGF-β signaling (10, 14, 18–22). Some lytic proteins, notably the anti-apoptotic Bcl-2 homologue BHRF1 and the early protein BALF1, are expressed at low levels even during latency. BHRF1 promotes Wp-restricted Burkitt lymphomagenesis (23, 24), whereas BALF1 enhances *in-vivo* transformation by increasing cell survival, thereby blurring the latency-lytic divide (25).

Lytic proteins also shape the tumour microenvironment. The transactivator ZTA induces secretion of pro-angiogenic cytokines such as IL-6, IL-8, IL-10, and IL-13 (12, 26). BALF1 and BHRF1 suppress mitochondrial apoptosis (24), whereas BARF1 and the viral IL-10 homolog BCRF1 impede apoptotic signaling and downregulate receptors for IFN-γ, IL-8, and IL-17 (27, 28). In parallel, several lytic proteins—including BNRF1, BALF3, BGLF4, and BGLF5—destabilize the host genome. BNRF1 induces centrosome amplification, BALF3 disrupts viral DNA packaging, BGLF4 interferes with cyclin D1 and ZBRK1 promoter function, and BGLF5 both triggers DNA damage and inhibits its repair, collectively accelerating genomic instability and mutation accumulation (29–33).

Despite its potent immunogenicity—illustrated by massive T-cell expansion during infectious mononucleosis (34)—EBV-induced widespread methylation of host and viral genomes fosters persistence and oncogenesis (35). Concurrently, epigenetic remodeling—particularly CpG island hypermethylation—silences host tumor suppressor genes and facilitates viral persistence, as exemplified in EBVaGC, where extreme methylation coexists with PIK3CA mutations and PD-L1/PD-L2 amplification (36–38).

The clinical and biological significance of EBV positivity varies considerably across cancer types. In some, like endemic BL and NKTCL, EBV is a defining feature present in nearly all cases. In others, such as gastric cancer and cHL, it delineates a distinct molecular and clinical subtype, often with unique therapeutic vulnerabilities. A fascinating and clinically critical commonality among many EBV-associated tumors is their “immune-hot” yet functionally suppressed TME, which paradoxically renders them promising candidates for immunotherapy, despite their potent immune evasion capabilities.

This review systematically compares and contrasts the epidemiological patterns, viral latency programs, and oncogenic mechanisms of major EBV-associated malignancies. A central theme is to identify convergent oncogenic pathways and shared immune vulnerabilities—such as NF-κB activation, epigenetic silencing, HLA down-regulation, and checkpoint activation—that transcend individual tumor types. Ultimately, we seek to provide a conceptual framework for developing pan-tumoral, EBV-directed therapeutic strategies.

## 2 Nasopharyngeal carcinoma

### 2.1 Epidemiology and incidence

Nasopharyngeal carcinoma (NPC) is a malignant tumor of epithelial origin that develops from the nasopharyngeal mucosa, with a predilection for the roof and lateral walls, notably the pharyngeal recess. The disease exhibits a striking geographic distribution, with high incidence rates in East and Southeast Asia (e.g., southern China) and North and East Africa (39). According to recent estimates from the International Agency for Research on Cancer (IARC), NPC ranks as the 23rd most common cancer globally, with 120, 416 new cases annually, accounting for approximately 0.6% of all new cancer diagnoses. It caused an estimated 73, 476 deaths, representing about 0.8% of global cancer mortality (40). According to 2022 statistics from China’s National Cancer Center, nasopharyngeal carcinoma (NPC) showed a marked male predominance. The annual tally reached 51, 000 new diagnoses, yielding an overall incidence of 5.08 cases per 100, 000 individuals. This disparity is reflected in the age-standardized rates, which were 3.39 for males compared to 1.33 per 100, 000 for females (41). The association linking Epstein-Barr virus (EBV) to nasopharyngeal carcinoma (NPC) was put forward in 1969 in a study by de Schryver et al., who observed elevated EBV-related antibody titers in patient sera (42). This link was substantiated in 1970 when zur Hausen et al. provided direct histological evidence by detecting EBV DNA in NPC biopsy specimens (43).

### 2.2 EBV-driven oncogenesis

Within nasopharyngeal carcinoma, Epstein-Barr virus primarily adopts a latency II program. This state is defined by the production of viral proteins including EBNA1, LMP1, and LMP2, as well as non-coding transcripts like EBERs and BART miRNAs (44). Some studies have also reported epithelial tumor-associated expression of BARF1 (45). Somatic alterations in NPC cooperate with EBV latent genes, particularly LMP1, to establish an oncogenic network centered on constitutive NF-κB activation (46). As a key latent oncoprotein, LMP1 activates multiple signaling cascades, including NF-κB, STAT3, and mTORC1, thereby promoting glycolysis, survival, and invasion of malignant cells (47). EBV, notably through LMP1, can also upregulate epigenetic enzymes like DNMT1 and DNMT3B, leading to hypermethylation and silencing of multiple tumor suppressor gene promoters, including PTEN, CDH1, DAPK, and RASSF1A (48–51). Although EBV-associated gene fusions are relatively uncommon, they are identified in a minority of patients and may represent targets for therapeutic intervention. Several EBV latent and lytic factors, including LMP1, BART miRNAs, EBNA1, and BALF3, have been shown to promote the occurrence of DNA damage, disrupt repair processes, and enhance chromosomal instability, thereby indirectly facilitating fusion events (31, 52, 53).

### 2.3 HLA and EBV immune evasion (host genetic susceptibility)

Host genetic susceptibility to NPC is strongly influenced by HLA-mediated presentation of EBV epitopes. Genome-wide association studies (GWAS) and high-resolution HLA typing have identified HLA-A\02:07 and HLA-B\46:01 as risk alleles, whereas HLA-A\11:01 is protective (54, 55). Importantly, high-risk EBV strains can interact with specific HLA alleles, further amplifying disease susceptibility. Earlier molecular and population-based evidence suggests that EBV can evade prevalent protective alleles (such as HLA-A\11) through epitope variation, thereby weakening antigen presentation and cytotoxic T-cell surveillance (56). Additionally, non-classical MHC-related genes such as TRIM31 and TRIM39 have been implicated in modulating innate immunity against EBV, further shaping overall susceptibility (55).

EBV promotes immune evasion in NPC through multiple mechanisms. In antigen processing and presentation, the Gly-Ala repeat (GAr) sequence of EBNA1 inhibits proteasome-mediated degradation, reducing peptide presentation via MHC-I (57, 58). Histological observations also show downregulation of HLA-I and TAP1/2, further impairing CD8^+^ T-cell recognition. At the immune checkpoint level, LMP1 directly upregulates both membrane-bound and soluble PD-L1, while EBV-encoded microRNAs such as BART11/17-3p also enhance PD-L1 expression, collectively suppressing T-cell function (59). In innate immunity, BART7 can downregulate MICA/MICB or inhibit their upstream regulatory axis, reducing NK cell–mediated cytotoxicity. Additionally, BARF1 functions as a decoy receptor for M-CSF, suppressing macrophage differentiation and activation, thereby fostering an immunosuppressive microenvironment (60).

EBV also remodels cell death pathways in NPC, contributing to therapeutic resistance. LMP1 upregulates BNIP3 and releases Beclin-1, inducing “protective autophagy” and conferring radioresistance (61). Moreover, EBV infection activates the p62–Keap1–NRF2 axis, leading to upregulation of GPX4 and SLC7A11, which renders tumor cells resistant to ferroptosis and more tolerant to chemotherapy. These data suggest that exploiting ferroptosis may address a key therapeutic vulnerability in Epstein-Barr virus-driven nasopharyngeal malignancies (62).

### 2.4 Tumor microenvironment

The immune landscape of nasopharyngeal carcinoma is composed of a heterogeneous array of cellular compartments. This ecosystem encompasses stromal and immune subsets such as cancer-associated fibroblasts (expressing COL1A1, COL3A1, FGF7, MME), endothelial cells (marked by FLT1, VWF, PECAM1), and epithelial cells (EPCAM, KRT5, KRT18, KRT19). Immunologically, it includes mast cells (TPSAB1^+^), myeloid-lineage cells (CD14^+^, ITGAX/CD11C^+^), B lymphocytes (CD19^+^, MS4A1^+^), and T/NK cells, which are identified by markers including PTPRC, CD3D/E, CD4, CD8A, FCGR3A, NCAM1, and KLRF1 (63, 64). Among these, T/NK cells and B cells represent the predominant immune components (63).

#### 2.4.1 Innate immunity in NPC

NK cells in NPC exhibit significant functional impairment (65), with downregulation of cytotoxic effector molecules like PRF1 and GZMB and upregulation of inhibitory receptors including TIGIT and LAG3 (66). Single-cell analyses classify NK cells into three subsets (NK1—NK3), of which the NK3 subset expresses ZNF683, indicating a tissue-resident phenotype, but concurrently shows high levels of TIGIT and LAG3, suggesting an exhausted state with pronounced functional impairment (67). These findings highlight TIGIT blockade as a potential therapeutic strategy to restore the cytotoxic activity of both NK and T cells.

Within the NPC tumor microenvironment (TME), monocytes and macrophages display bidirectional plasticity. Single-cell transcriptomic studies have identified multiple myeloid subpopulations, with C3-C1QA^+^ and C2-APOE^+^ macrophages predominating in normal tissues and lymph nodes. Notably, C1QA^+^ macrophages are particularly associated with immune regulation and VEGFA signaling pathways. This subpopulation shows significant expansion during tumor progression and has been demonstrated to contribute to immune evasion in metastatic esophageal squamous cell carcinoma (68, 69). By comparing primary lesions with metastatic lymph nodes, it was found that mononuclear/macrophages in primary lesions were widely upregulated in anti-inflammatory, pro-inflammatory, antigen presentation and chemotaxis related procedures, while these functions were widely inhibited in metastatic lymph nodes, suggesting that myeloid cells actively promote lymph node metastasis of nasopharyngeal carcinoma by establishing immunosuppressive microenvironment. Another study revealed strong activation of interferon-α/γ signaling in NPC macrophages, while monocytes are enriched for angiogenesis, NF-κB—mediated TNF-α signaling, and hypoxia-related pathways, with high expression of S100A8/A9, collectively indicating a pro-inflammatory and pro-tumorigenic potential (70, 71). Conversely, evidence also supports antitumor functions. Regulatory network analysis revealed similar transcription factor profiles between monocytes and macrophages, indicating local differentiation within the TME (72). In this process, transcription factors NR1H3 and TFEC are key drivers of monocyte-to-macrophage differentiation and maturation; their high expression is associated with lineage progression and correlates with improved patient prognosis (73, 74). Thus, the role of myeloid cells in NPC is not uniform but represents a dynamic balance between pro-tumor and antitumor states, shaped by transcriptional regulators and microenvironmental cues.

Meanwhile, dendritic cells (DCs) in NPC also display marked functional heterogeneity. Liu et al. (64) identified a DC_C3_LAMP3 subset characterized by reduced antigen-presenting capacity, with GO and KEGG analyses revealing significant downregulation of immune activation–related genes, suggesting a phenotype skewed toward immune regulation and tolerance. These cells are thought to suppress T-cell activation, thereby attenuating antitumor immunity. Notably, LAMP3^+^ DCs have been reported in multiple other cancers, where they exhibit strong migratory, activation, and maturation properties (75, 76), indicating a potential conserved role in shaping an immunosuppressive TME in NPC.

#### 2.4.2 Adaptive immunity in NPC

Lymphoid cells in the NPC microenvironment undergo profound functional reprogramming, with T cells as central effectors. Single-cell transcriptomic studies reveal enrichment of exhausted T cells, regulatory T cells (Tregs), and effector T cells within tumor tissues, whereas naïve and central memory T cells are predominantly located in non-malignant regions. Pathway analyses indicate that NPC-derived T cells exhibit pronounced type I/II interferon response activation, suggesting chronic antigen stimulation, in contrast to non-malignant T cells primarily regulated by the NF-κB pathway (77). Notably, exhausted T cells, despite high expression of inhibitory receptors like PD-1 and LAG3, retain partial cytotoxic activity, indicating a dynamic transition from effector to exhaustion. Liu et al. (64) identified four distinct Treg subclusters by single-cell transcriptomics, among which the Treg_C4_TNFRSF4 subset, characterized by high expression of TNFRSF4, TNFRSF9, and CCR8, along with inhibitory molecules like CTLA4, TIGIT, and HAVCR2, was deemed the most suppressive. This subset is highly enriched in NPC and closely associated with immune evasion and disease progression (78, 79). Furthermore, terminal-stage Tregs not only display strong proliferative potential but also utilize lactate metabolism to sustain their function while inducing PD-1 expression, thereby further dampening effector T-cell cytotoxicity (63, 80, 81).

B cells in NPC play a complex, dual role, with their functions evolving during disease progression. In early stages, they contribute to antitumor immunity through antigen presentation and antibody production (82, 83). Tumor-infiltrating B cells are often organized into tertiary lymphoid structures (TLSs) with Tfh cells, undergoing germinal center (GC) reactions to generate memory B cells and plasma cells (84). These plasma cell–derived antibodies can mediate antibody-dependent cellular cytotoxicity (ADCC) or phagocytosis (ADCP), indirectly activating NK cells and macrophages (85, 86). However, in advanced NPC, B-cell function declines, evidenced by a reduction in GC B cells and a decreased rate of clonal transition to plasma cells. Immunohistochemistry confirms lower IgA and IgG levels in late-stage disease, while transcriptomic analyses reveal downregulation of key genes involved in BCR signaling, protein transport, and peptide biosynthesis in plasma cells, impairing antibody synthesis. Thus, although plasma cell numbers may remain stable, their antibody production capacity and quality diminish with tumor progression. Notably, certain B-cell subsets in NPC also exhibit immunosuppressive features; for instance, regulatory B cells (Bregs) expressing PD-L1 or secreting IL-10 can suppress T-cell effector functions and potentially disrupt the Tfh-B cell axis, undermining GC responses (87). Collectively, these findings indicate that B cells in NPC have a stage-dependent role, promoting antitumor immunity early but undergoing functional impairment or adopting immunosuppressive phenotypes that facilitate immune evasion later.

#### 2.4.3 Tumor microenvironment in NPC

Malignant epithelial cells in NPC are characterized by high proliferative and invasive capacity, with elevated cell cycle activity strongly associated with poor prognosis. Common genomic alterations include deletions of 3p, 9p, 11q, 14q, and 16q, as well as amplification of 12q (70, 88, 89). Transcriptomic analyses have further identified a KIF18B-high subgroup associated with with an immunosuppressive phenotype, activation of epithelial-mesenchymal transition, increased m6A RNA methylation, and diminished efficacy of immunotherapeutic interventions. These cells are accompanied by increased Treg infiltration but reduced CD8^+^ T cells and macrophages, while activating immunosuppressive pathways such as NOTCH and WNT, suggesting a critical contributor to NPC progression and immune evasion (90–93). A distinct subset of “fibroblast-like malignant cells” has also been reported, characterized by COL1A1 expression and chromosomal copy number variations. These cells exhibit reduced antigen-presenting capacity and enhanced immune evasion and are considered potential cancer stem cell (CSC)-like populations (94). Furthermore, EBV DNA–positive samples are enriched in IFN-α/γ, hypoxia, p53, and NF-κB/TNF-α signaling pathways, underscoring the role of virus-associated signaling in shaping the TME (95–97).

**Figure 1:**
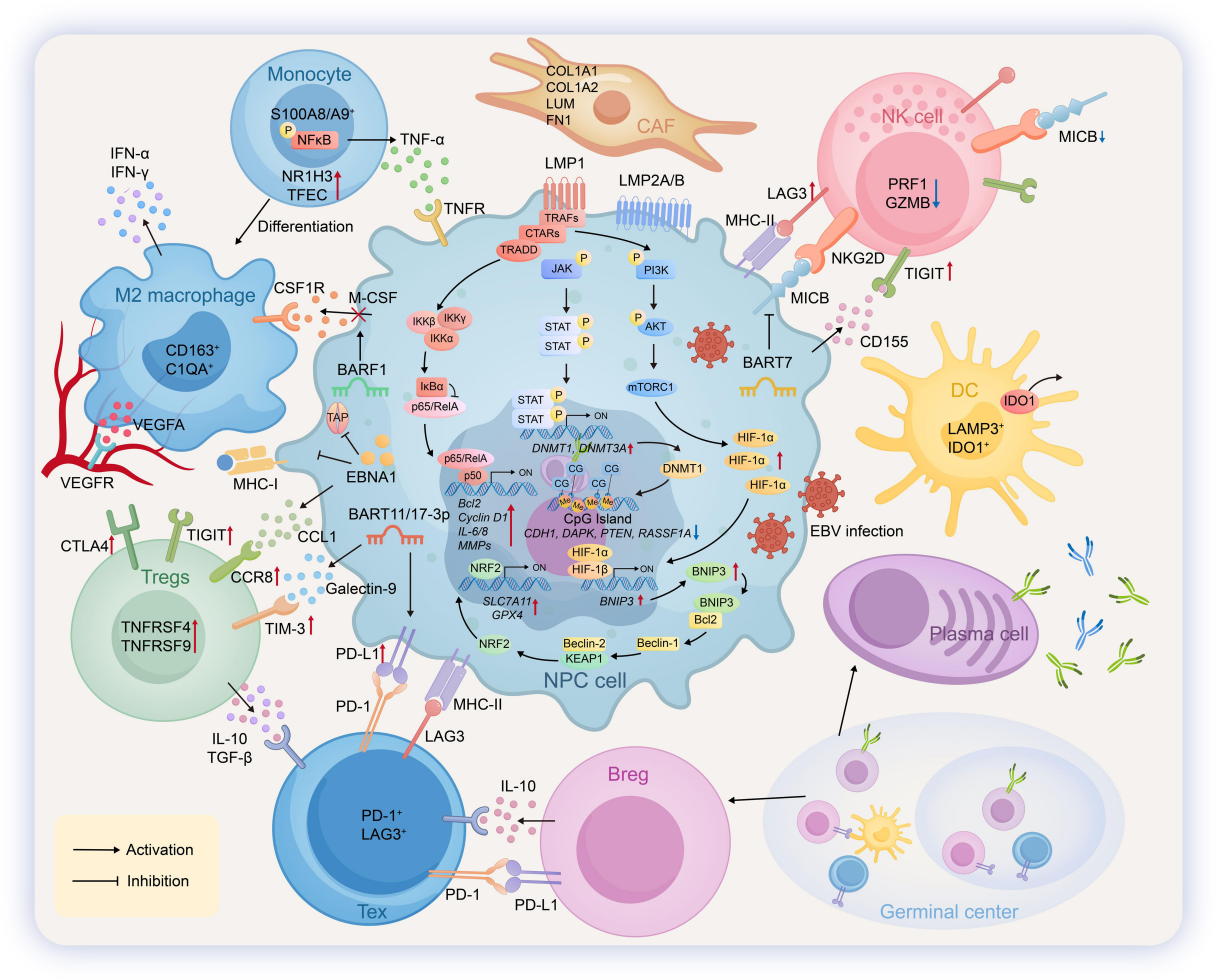
Pathogenic mechanisms and immune microenvironment in NPC. In nasopharyngeal carcinoma (NPC), EBV establishes a type I/II latency, expressing LMP1, LMP2, and EBNA1. LMP1 drives oncogenesis by activating NF-κB (promoting proliferation/inflammation), JAK/STAT (inducing epigenetic silencing), and PI3K/AKT/mTORC1 (inhibiting ferroptosis) pathways. For immune evasion, NPC cells express PD-L1/MHC-II and secrete CCL1/Galectin-9. The NPC tumor microenvironment is characterized by extensive infiltration of immune cells with altered functions. Natural killer (NK) cells exhibit impaired cytotoxicity, partly due to downregulated MICB. Monocytes secrete TNF-α and can differentiate into pro-angiogenic M2 macrophages that release VEGFA. Regulatory T cells (Tregs) highly express inhibitory molecules such as CTLA4 and TIGIT. Exhausted T cells (Tex) are marked by high levels of inhibitory receptors including PD-1, TIM-3, and LAG-3. Regulatory B cells (Bregs), differentiated from germinal center naïve B cells, secrete immunosuppressive cytokines like IL-10 to modulate T-cell function. Dendritic cells (DCs) express elevated levels of IDO1 and LAMP3 and are involved in antigen presentation and fostering immune tolerance. Cancer-associated fibroblasts (CAFs) secrete extracellular matrix components (e.g., COL1A1/A2, LUM, FN1) and interact with NPC-derived TNF-α via TNFR, collectively remodeling the structural and signaling landscape of the tumor microenvironment.

Cancer-associated fibroblasts (CAFs) are pivotal in NPC progression, regulating tumor cell proliferation, migration, angiogenesis, and immune evasion (98–100). In NPC, fibroblasts are markedly enriched and specifically overexpress multiple extracellular matrix (ECM)-related genes, including COL1A1, COL1A2, LUM, and FN1 (77). These ECM components interact with integrin receptors to mediate cell adhesion, migration, and survival, thereby promoting dynamic ECM remodeling and activating integrin signaling pathways that drive tumor progression and immunosuppression. Consequently, targeting integrins and their interactions with ECM molecules represents a potential therapeutic strategy (**Figure 1**).

### 2.5 Potential targets

Single-cell transcriptomic data reveal considerable heterogeneity within the NPC TME, suggesting potential targets for immunotherapy. For instance, elevated expression of immune checkpoint molecules in T-cell subsets indicates a predominantly immunosuppressive state. Although PD-1/PD-L1 blockade has shown clinical benefit in a subset of patients, the overall response rate remains only 20–30% (101–103). Recent studies suggest that therapeutic response is closely associated with baseline TME features: patients achieving complete remission often display lower Treg levels, higher CD8^+^ T-cell infiltration, and upregulation of IFNG and antigen-presentation–related genes (104). Conversely, high SPP1 expression correlates with an immunosuppressive milieu and poor outcomes (105, 106). During therapy, the proportion of CD8^+^ T cells within tumor regions increases, while Tregs, exhausted CD8^+^ T cells, and malignant cells decline, indicating immune microenvironment repolarization (107). However, some patients acquire resistance through upregulation of alternative inhibitory molecules, such as CD47, underscoring the need to optimize combination immunotherapeutic strategies.

### 2.6 mRNA vaccines

Owing to rapid design, high-level expression and an favourable safety profile, mRNA vaccines have attracted increasing attention for the treatment of EBV-positive NPC. Pre-clinical and clinical efforts have centred on viral antigens expressed during EBV latency, notably the latent membrane proteins LMP1 and LMP2A/B and the nuclear antigens EBNA1 and EBNA3A. Among these, LMP2A—stably expressed in NPC tissue and inherently immunogenic—has emerged as the principal vaccine target.

In pre-clinical models, a liposome-encapsulated LMP2 mRNA vaccine (LPX-mLMP2) (108) promoted dendritic-cell maturation and elicited LMP2-specific CD8^+^ T-cell responses that substantially inhibited LMP2-positive tumour growth. A multi-antigen mRNA vaccine encoding truncated forms of EBNA1, EBNA3A and LMP2A similarly induced robust cellular and humoral immunity and prolonged survival (109). WGc-043, an EBV mRNA vaccine whose antigenic sequences were selected by artificial intelligence and formulated with built-in immuno-enhancers, has entered the clinic. In a phase I trial(NCT05714748) (110) enrolling patients with EBV-positive recurrent/metastatic NPC, WGc-043 achieved a disease-control rate of 66.67% and an objective response rate of 16.67%; 91.7% of participants exhibited a decrease in circulating EBV DNA. The vaccine was well tolerated and provided preliminary evidence of efficacy.

### 2.7 Cellular therapy

TCR-engineered T-cell (TCR-T) therapy, capable of targeting intracellular antigens, has become a central pillar of EBV-positive NPC cellular therapy. TCRs are preferentially directed against latent viral antigens—most commonly LMP1, LMP2 and EBNA1. YT-E001, an EBV-specific TCR-T product, is currently undergoing evaluation in a Chinese phase II trial (NCT03648697) for relapsed/metastatic EBV-positive NPC; interim analyses indicate robust EBV-directed immune re-activation with acceptable toxicity.

Although CD19-directed CAR-T cells have transformed haematological oncology, their extension to EBV-positive solid tumours is constrained by heterogeneous antigen expression and a profoundly immunosuppressive microenvironment. Early-phase studies exploring CAR-T cells targeting EpCAM or LMP2 are underway (NCT02980315, NCT05587543), but definitive efficacy and safety data are pending.

NK-cell-based immunotherapy is also under investigation. EBV skews tumour-associated macrophages toward an M2 phenotype and up-regulates inhibitory ligands such as PD-L1, thereby evading NK surveillance (111, 112). Combined blockade of F3 or genetic deletion of B7-H3 together with PD-L1 antagonism re-invigorates NK cytotoxicity and yields synergistic tumour control in murine NPC models (113).

## 3 EBV-associated intrahepatic cholangiocarcinoma

### 3.1 Epidemiology and incidence

A rare and distinct clinicopathological variant of intrahepatic cholangiocarcinoma, known as EBV-associated intrahepatic cholangiocarcinoma (EBVaICC), has emerged in recent years. A meta-analysis comprising 15 studies and 918 cases reported an overall EBV infection rate of 23% (95% CI: 13%–33%, I² = 95.7%, P < 0.001) among liver cancer patients (114). EBV-associated lymphoepithelial carcinoma (LEC) of the liver was first reported in 1996, when Hsu et al. identified EBV genomes in an intrahepatic lymphoepithelioma-like tumor (115). Later, in 2001, Chen et al. confirmed EBV presence by EBER *in situ* hybridization in two cases of LEL-ICC, reinforcing the association between EBV and this histologic subtype (116). At present, no independent epidemiological statistics exist for EBVaICC incidence or mortality; such data are usually included within overall ICC figures, and the incidence of ICC has been rising in recent years (117). EBVaICC cases reported to date are concentrated in East and Southeast Asia, mirroring the geographical distribution of EBV-associated nasopharyngeal carcinoma and gastric carcinoma, and are exceedingly rare in Western populations. In southern China, patients are predominantly female (male-to-female ratio ≈ 1:3), present at a relatively young age (median 46.5 years), frequently have concurrent HBV infection (≈ 50%), but exhibit a low prevalence of liver cirrhosis (118).

### 3.2 EBV-driven oncogenesis

In EBV-associated intrahepatic cholangiocarcinoma (EBVaICC), most studies indicate a type I latency pattern, characterized by EBER and EBNA1 expression, with absent or variable LMP1/2 (118). Although EBV has been clearly shown in NPC and lymphomas to drive proliferative signaling through LMP1/LMP2 and to inhibit tumor suppressor pathways, direct experimental evidence in liver cancers (HCC/ICC) remains limited. To date, only indirect studies suggest that EBV may contribute to hepatocarcinogenesis by facilitating replication of other viruses (e.g., HCV) and sustaining chronic inflammation (119, 120). Systematic investigations into host genetic susceptibility, particularly HLA alleles in EBVaICC, are currently lacking. While pathological reports suggest a close relationship between EBVaICC and its immune microenvironment, no definitive HLA association has been established. In terms of immune evasion, the best-documented mechanism in EBVaICC is an enhancement of PD-1/PD-L1 signaling. A recent cohort work by Huang et al. (2025) (121) found that EBVaICC patients receiving anti–PD-1 therapy had significantly improved overall survival (OS) and progression-free survival (PFS). Using newly established EBV^+^ iCCA cell lines, the study further demonstrated that EBV activates the cGAS–STING pathway, upregulating MHC-I and increasing CXCL10 secretion, thereby enhancing tumor immunogenicity (121). Additionally, during type I latency, the EBNA1 protein uses its Gly–Ala repeat (GAr) domain to suppress its own mRNA translation, reducing MHC-I–mediated antigen presentation and facilitating immune escape (57, 122). The roles of apoptosis, necroptosis, and autophagy in EBVaICC remain poorly defined, with current understanding largely extrapolated from other EBV-related cancers. Future studies using EBV^+^ iCCA models should clarify whether EBV similarly manipulates these cell death pathways to promote survival and immune evasion in iCCA.

### 3.3 Immune profiles of EBVaICC

**Figure 2:**
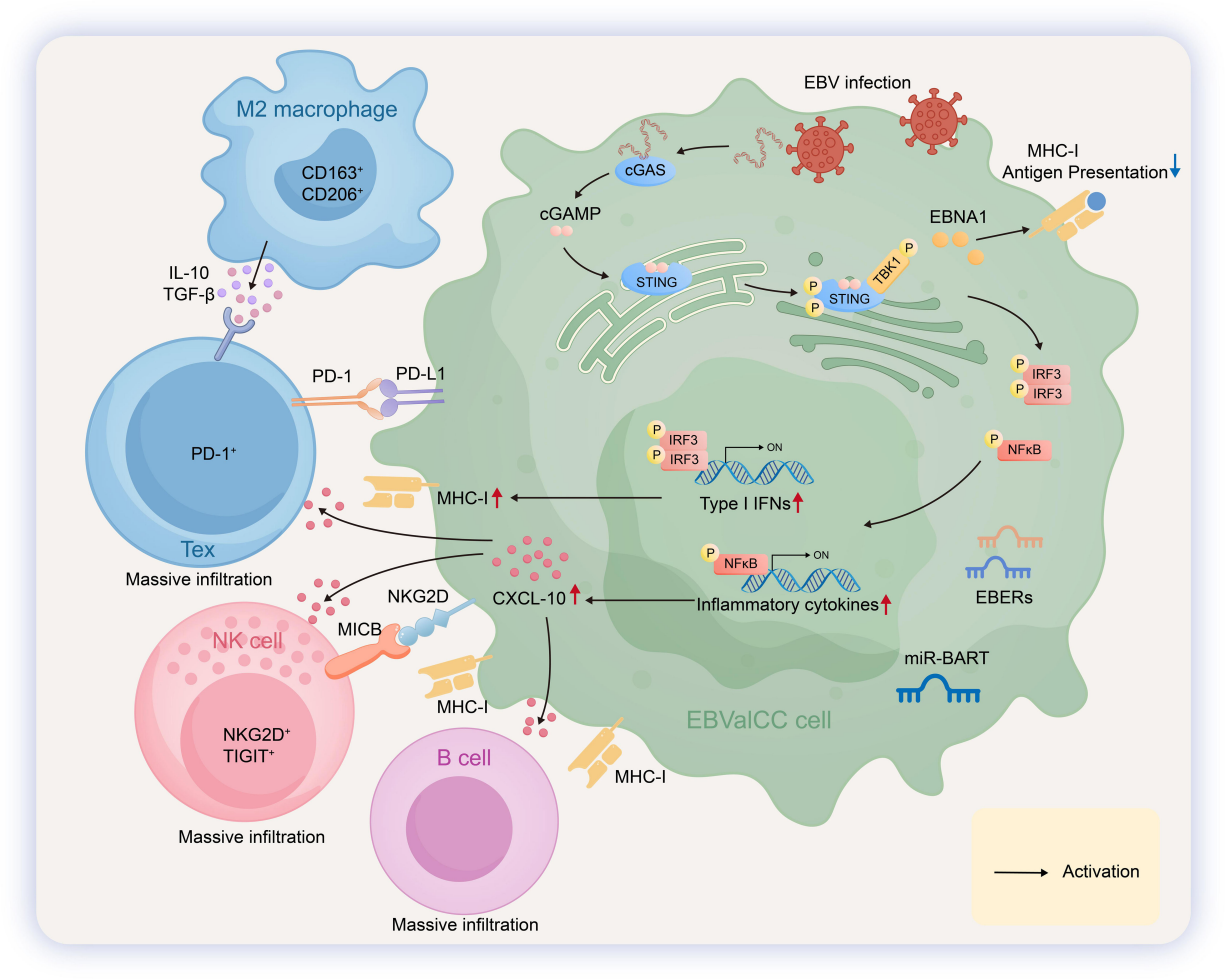
Pathogenic mechanisms and immune microenvironment in EBVaICC. In EBV-associated intrahepatic cholangiocarcinoma(EBVaICC), EBV establishes a type I latency, expressing EBERs,EBNA1. Upon EBV infection, cytoplasmic viral DNA is sensed by cGAS, which catalyzes the synthesis of cyclic GMP-AMP (cGAMP). cGAMP then activates the adapter protein STING. Following its phosphorylation by TBK1, STING facilitates the phosphorylation of IRF3 and NF-κB, leading to the upregulated expression of type I interferons and inflammatory cytokines such as CXCL10, respectively. EBV-associated intrahepatic cholangiocarcinoma (EBVaICC) possesses an immune-”hot” tumor microenvironment. Natural killer (NK) cells with high expression of NKG2D and TIGIT extensively infiltrate the tumor. They recognize target cells via the NKG2D-MICB axis and through the loss of MHC-I molecules (missing-self recognition). Exhausted T cells (Tex) exhibit elevated PD-1 expression. M2-polarized macrophages contribute to immune regulation by secreting IL-10 and TGF-β. B cells are also abundantly present within the tumor lesions and engage in interactions with MHC-I molecules.

Among cholangiocarcinoma subtypes, lymphoepithelioma-like cholangiocarcinoma (LEL-CC) shows the strongest EBV association, with reported positivity rates between 55% and 73.1% (118, 123). In a cohort of 303 intrahepatic cholangiocarcinoma (ICC) cases, EBVaICC accounted for about 6.6%, with a predilection for younger and female patients. Clinically, EBVaICC typically presented as solitary tumors, often with concomitant HBV infection, and less frequently with underlying cirrhosis; the LEL subtype was most common (124). Notably, EBV has been detected only in ICC and not in perihilar (pCCA), distal (dCCA), or combined hepatocellular–cholangiocarcinoma (cHCC-CCA), suggesting a site-specific tropism reminiscent of EBV-associated gastric carcinoma (125). Molecularly, EBVaICC displays distinct immune characteristics. Comparative analyses by Chiang et al. revealed that EBV-positive LEL-CC, compared to EBV-negative ICC, showed significant enrichment of adaptive immune response pathways, with higher activation of multiple immune populations—including CD45^+^ cells, B cells, T cells, Th1 cells, CD8^+^ T cells, and exhausted CD8^+^ cells. mRNA expression of PDCD1 (PD-1) and CD274 (PD-L1) was also markedly elevated, indicating strong immune checkpoint activation (126). This profile resembles that of EBV-positive gastric cancer and supports the potential efficacy of immunotherapy in EBVaICC. Recent clinical evidence further corroborates this: among ICC patients receiving PD-1 inhibitors, those with EBVaICC had significantly longer overall survival and higher response rates compared to EBV-negative cases. Circulating EBV-DNA levels decreased with treatment response and increased upon progression, suggesting its utility as a dynamic biomarker for monitoring therapeutic efficacy (**Figure 2**) (127).

### 3.4 Potential targets

EBV-associated intrahepatic cholangiocarcinoma (EBVaICC) lacks a single dominant driver mutation; however, the virus itself and its intricate interactome with the host provide multiple actionable nodes. Beyond the validated high PD-L1 expression and “hot” immune microenvironment that underpin the clinical utility of PD-1/PD-L1 blockade, constitutive expression of the latent membrane proteins LMP1/2 and the nuclear antigen EBNA1 (128), not only serves as direct targets for T-cell or antibody–drug conjugate therapies, but also furnishes a ready-made viral antigen repertoire for mRNA vaccine design. Preliminary studies indicate that mRNA vaccines encoding LMP2A or EBNA1 can elicit EBV-specific CD8^+^ T-cell responses *in vitro*, and are therefore poised to be integrated with immune-checkpoint inhibitors in future “vaccine-immunotherapy” sequential regimens. Concurrently, LMP1-driven PI3K/AKT/mTOR and JAK/STAT3 signaling cascades have been shown to be susceptible to selective small-molecule inhibition, laying the theoretical groundwork for a dual viral-and-signal blockade strategy. Based on the prevalence of targetable FGFR2 fusions and IDH1 R132C in unselected iCCA cohorts, approximately 15% of EBVaICC patients are anticipated to harbor these alterations (129), allowing for the concurrent use of FGFR inhibitors (e.g., pemigatinib) or IDH1 inhibitors (ivosidenib) and thus shaping a “virus-plus-tumor” bifocal targeting landscape. Within this framework, infusion of EBV-specific cytotoxic T lymphocytes (CTLs) or CAR-T cells directed against LMP1/2 or EBNA1 may eradicate residual virus-positive tumor cells, while epigenetic agents such as HDAC inhibitors—by up-regulating viral antigen expression—are expected to potentiate the synergistic effects of mRNA vaccines and cellular therapies. Collectively, the therapeutic blueprint for EBVaICC is evolving from a “single-target” paradigm to a three-dimensional “virus–host–mutation” network, with mRNA vaccines and cell-based immunotherapies emerging as pivotal components of precision strategies guided by dual molecular classification.

## 4 EBV-associated gastric cancer

### 4.1 Epidemiology and incidence

Globally, gastric cancer constitutes a major health burden and is one of the most common cancers. Data from the 2022 Global Cancer Statistics indicate that it holds the fifth position for both global incidence and cancer-related deaths, accounting for roughly 660, 000 fatalities each year. Established contributors to its development are infection with Helicobacter pylori, older age, excessive salt consumption, and inadequate intake of fruits and vegetables (41). Notably, ebv infection contributes to the etiology of roughly 10% of global gastric cancer burdens (130). EBV-associated gastric cancer (EBVaGC) exhibits distinct epidemiological patterns, with incidence rates typically higher in males than females (male-to-female ratio ∼2:1 to 3:1), potentially due to sex-based differences in immune response, exposure routes, and lifestyle factors. Geographically, EBVaGC is more prevalent in East Asia (including China, Japan, and South Korea) and Latin America, accounting for approximately 8%-15% of all gastric cancer cases in China and 10%-15% in Japan and South Korea (131, 132).

### 4.2 EBV-driven oncogenesis

The circular EBV episome resides and is maintained within the nucleus of the host cell, adopts chromatin structure, and undergoes extensive methylation, thereby evading immune surveillance and maintaining latency. In EBVaGC, the virus exhibits type I–II latency, expressing EBNA1, LMP2A, BARF1, and EBERs. EBNA1 ensures viral DNA replication and segregation, while LMP2A, BARF1, and EBERs act synergistically (133). LMP2A functions as the central driver that constitutively activates NF-κB, PI3K/AKT, JAK/STAT3, Wnt/β-catenin, and Notch signaling axes: on the one hand, it phosphorylates and degrades IκB, enabling nuclear translocation of NF-κB and upregulation of anti-apoptotic genes including Bcl-2 and survivin (134, 135); on the other hand, it inhibits GSK-3β to stabilize β-catenin, promotes EMT, and enhances tumor cell migration and invasion (136). In addition, LMP2A phosphorylates STAT3 to up-regulate the methyltransferases DNMT1/3a, establishing a CIMP-high phenotype (CpG-island methylator phenotype) across 886 gene promoters and broadly silencing tumor-suppressor genes including p16INK4A, p15, p14ARF, PTEN, APC, CDH1, and Rec8 (133, 137), resulting in dysregulation of the cell cycle, apoptosis resistance, EMT, and immune evasion. A recent study further revealed that EBV sustains proliferation and survival by activating the Hippo–YAP pathway; via the cGAS–STING axis, EBV up-regulates OLFM4—an extracellular inhibitor of Hippo kinases—thereby enhancing YAP-driven oncogenic growth (138).

### 4.3 HLA and EBV immune evasion

The HLA profile and immune-evasion machinery of EBV-associated gastric cancer (EBVaGC) jointly sculpt its paradoxical “hot-yet-immunosuppressed” phenotype. On the one hand, the tumors ubiquitously transcribe all classical HLA-II loci (HLA-DP/DR/DQ α/β chains) together with their master regulators CIITA and RFX5, while the rate of complete HLA-I loss is only ~23%—significantly lower than the 52% observed in microsatellite-unstable (MSI) gastric cancers—indicating that CD8^+^ T-cell antigen presentation remains largely intact (139, 140). On the other hand, 9p24.1 amplification and viral proteins EBNA1/LMP2A directly activate PD-L1 transcription through the JAK2-STAT1/IRF-1 or canonical NF-κB axes, thereby dampening CD8^+^ T-cell cytotoxicity and promoting tumor-specific T-cell apoptosis and Treg differentiation (141, 142). EBV-encoded miR-BART11 and BART17-3p reinforce this pathway by targeting FOXP1 and PBRM1 to relieve their repression of PD-L1, while TAP1—up-regulated by the same LMP2A-NF-κB signalling module—feeds forward via JNK-STAT1 to sustain PD-L1 over-expression, synergistically amplifying the immunosuppressive signal (143, 144). Beyond PD-L1, CTLA-4, LAG-3 and IDO1 are concurrently up-regulated in EBVaGC, further assisting tumor cells in evading immune surveillance (145). Although population studies suggest that HLA-A02 confers protection and A01 may increase susceptibility to EBV-related malignancies, consistent associations have not been established for EBVaGC itself (10, 146), implying that the immune-escape advantage of EBVaGC is driven primarily by complex virus–host interactions rather than by a single HLA allele.

### 4.4 Tumor microenvironment

Epstein-Barr virus-positive gastric cancer (EBVaGC) is established as a distinct clinicopathologic variant with unique features. In contrast to the typically immune-cold and T-cell–sparse microenvironment of EBV-negative gastric cancer (EBVnGC), EBVaGC exhibits extensive lymphocytic infiltration and an immune-hot, inflammatory TME (147). This microenvironment is enriched with CD8^+^ T cells, CD4^+^ T cells, and dendritic cells, all present at significantly higher densities than in EBVnGC (148, 149). The robust immune infiltration characteristic of EBVaGC underpins its increased sensitivity to immunotherapy, a key factor that contributes to its improved prognosis relative to EBVnGC (126, 148).

#### 4.4.1 Innate immunity in EBVaGC

In EBV-associated gastric cancer (EBVaGC), tumor-associated macrophages (TAMs) account for up to 22% of the tumor microenvironment (TME), representing the second-largest immune population after CD8^+^ T cells (150). Compared with EBV-negative gastric cancer, early-stage EBVaGC is dominated by CD68^+^ M1-polarized macrophages that produce high levels of TNF-α and IL-12, exhibit robust phagocytic and antigen-presenting capacity, and activate Th1 responses that restrain tumor growth. As disease progresses, the proportion of CD204^+^/CD163^+^ M2 macrophages increases; these cells secrete IL-10, TGF-β, and CCL17, thereby promoting angiogenesis and immune escape and conferring a worse prognosis (149). Clinically, a higher intratumoral density of total CD68^+^ macrophages correlates with larger tumor volume, whereas low M2 macrophage density is associated with prolonged patient survival (149).

Natural killer (NK) cells, although scarce in the EBVaGC TME, sustain potential anti-tumor activity through up-regulation of cytotoxic genes such as CD160 and GZMH (151). However, EBV can impair NK cytotoxicity via the LMP2A–PI3K/AKT–F3 axis, which induces platelet aggregation and generates an immunosuppressive milieu (152). Consequently, Approaches to augment NK-cell cytotoxicity—such as blocking the F3-mediated inhibitory axis or combining NK-cell adoptive transfer—may represent novel immunotherapeutic avenues for EBVaGC.

#### 4.4.2 Adaptive immunity in EBVaGC

In the tumor immune microenvironment of EBVaGC, T cells not only accumulate numerically but also exhibit a triad of high proliferation, high activation, and high exhaustion. Single-cell sequencing shows that the EBVaGC immune landscape is notably “hot, “ characterized by a dense co-infiltration of CD8^+^ T cells alongside CD20^+^ B cells and mature dendritic cells (DCs), all present at levels substantially exceeding those in EBV-negative gastric cancer (151), and T cells are highly clonally expanded. TCR sequencing indicates that 61–73% of T cells contain detectable TCRα/β chains, with 13–33% being hyper-expanded clones (100–500 copies), again suggesting persistent EBV antigen drive (148). Single-cell sequencing identifies a subset of EBV-specific ISG-15^+^CD8^+^ T cells that reside in a type-I-interferon-activated transitional precursor exhausted (Tpex) state; their baseline abundance predicts immunochemotherapy efficacy. In responders, these ISG-15^+^ CD8^+^ T cells undergo “clonal resurrection” after PD-1 blockade and differentiate into CXCL13^+^ effector T cells, ZNF683^+^ tissue-resident T cells, or GZMB^+^ cytotoxic T cells. CXCL13, a chemokine secreted by T follicular helper cells, recruits immune cells into the TME and is linked to favorable immunotherapy responses in multiple cancers, including gastric cancer. Clonal expansion of ISG-15^+^CD8^+^ T cells is associated with the emergence of CXCL13-expressing effector T cells, highlighting their role asdynamic mediators of treatment response and potential biomarkers for personalized immunotherapy (131, 148). In non-responders, however, this population undergoes terminal exhaustion due to sustained high LAG-3 expression. Pre-clinical and early-phase clinical trials show that targeting LAG-3 can reverse this exhausted state and achieve partial tumor remission, suggesting that LAG-3 is the most promising immune-checkpoint target after PD-1 in EBVaGC (147, 148).

The pronounced increase in B-cell and plasma cell abundance within EBVaGC, relative to EBV-negative cases, highlights their pivotal involvement in mounting both antiviral and antitumor immunity. B cells engage in extensive crosstalk with T cells and myeloid cells and exhibit dynamic changes during therapy (147, 148). Through secretion of cytokines such as IL-6 and IL-10, B cells modulate T-cell activity and may promote regulatory T-cell generation, fine-tuning immune activation within the TME (139, 148). Following treatment, B cells in EBVaGC show increased clonal expansion and BCR diversity, indicative of an active, adaptive immune response that may enhance antitumor immunity via antibody production or immune regulation (148). Single-cell analyses further reveal functional heterogeneity among B-cell subsets, with some exhibiting high proliferative activity and others specializing in antibody secretion. Histologically, tertiary lymphoid structures (TLS) in EBVaGC are enriched with CD19^+^ B cells colocalized with CD4^+^ and CD8^+^ T cells, facilitating B–T cell interactions that amplify T-cell effector functions and improve immunotherapy outcomes (148, 153). The prominence of B-cell and germinal center signatures in EBVaGC may partly explain its favorable response to immunotherapy, highlighting B cells as potential contributors to antitumor immunity.

#### 4.4.3 Tumor microenvironment in EBVaGC

**Figure 3:**
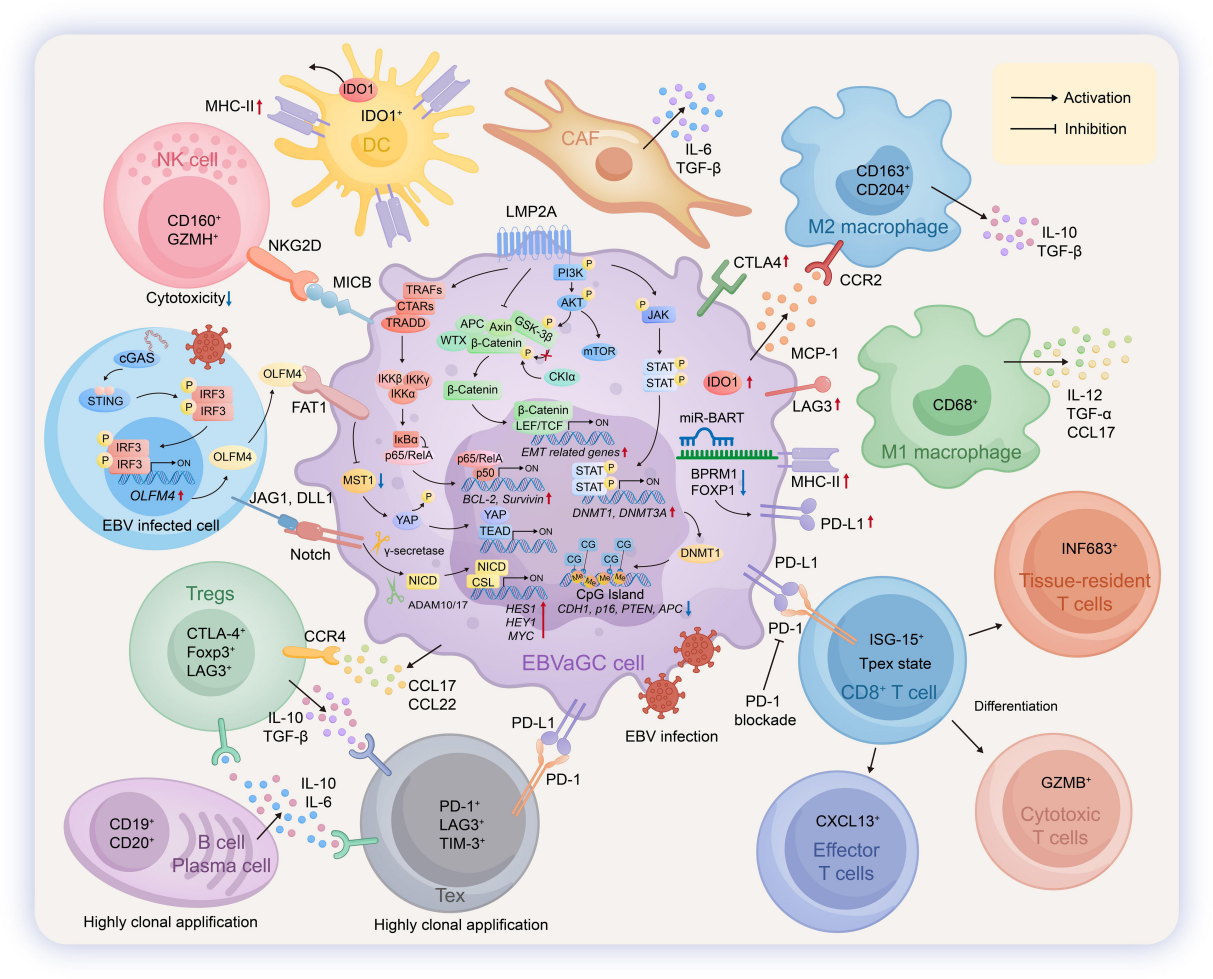
Pathogenic mechanisms and immune microenvironment in EBVaGC. In Epstein–Barr virus-positive (EBV+) extranodal NK/T-cell lymphoma (NKTCL), EBV establishes a type I/II latency. In NKTCL, EBV infection drives the malignant phenotype. The disease can be molecularly classified into three subtypes: MB (Latency I), HEA (Latency II), and TSIM (Latency II), each expressing distinct patterns of EBV latent proteins including EBNA1 and LMP1/2. The oncoprotein LMP1 activates the NF-κB pathway to modulate apoptosis, while EBNA1 enhances the expression of survivin via the JAK-STAT pathway. LMP2, in parallel, regulates the stability of survivin through the PI3K/PDK1/AKT signaling cascade. Furthermore, the binding of hepatocyte growth factor (HGF) to its receptor c-Met on NKTCL cells activates downstream pathways that drive tumor cell proliferation. To facilitate immune escape, NKTCL cells secrete dipeptidyl peptidase 4 (DPP4), which degrades the chemokines CXCL9 and CXCL10, thereby impairing the recruitment of T and NK cells to the tumor site. Helper T lymphocytes (HTLs) recognize antigens presented by MHC-II molecules (HLA-DR9/12/53) on NKTCL cells via their T-cell receptors (TCRs), participating in antigen presentation and immune regulation. Regulatory T cells (Tregs) engage with CD80/86 on NKTCL cells through CTLA-4 and secrete IL-10 and TGF-β to mediate immunosuppression. Exhausted T cells (Tex) are functionally suppressed upon interaction with PD-L1 on tumor cells via PD-1. T and NK cells are recruited to the microenvironment in response to CXCL9/CXCL10 signals. Tumor-associated macrophages (TAMs) contribute to the immunosuppressive milieu by secreting cytokines such as IL-10, IL-19, and VEGF. Dendritic cells (DCs) further reinforce immune suppression through the expression of indoleamine 2, 3-dioxygenase 1 (IDO1).

EBVaGC frequently exhibits a distinct “carcinoma with lymphocyte stroma” (CLS) subtype, in which neoplastic cells are encircled by dense lymphocytic infiltrates and abundant stromal components, implying that extracellular matrix (ECM) is pivotal for maintaining tumour architecture and facilitating signalling (154). Cancer-associated fibroblasts (CAFs) scattered throughout this ECM release multiple factors (e.g., TGF-β, IL-6) that drive invasion, metastasis and immune evasion (155). EBVaGC also expresses a spectrum of chemokines: CXCL9, linked to T-cell recruitment, may help establish an immune-hot micro-environment, whereas CCL18 is markedly up-regulated in the CLS subtype and probably modulates immune-cell chemotaxis and tumour–stromal crosstalk (156, 157). Additionally, cytokines such as the IFN-γ-inducible factor IDO1 contribute to immune regulation and suppression, while IL-6 and IL-10 propagate inflammation and further immune escape (156). Single-cell transcriptomic studies have identified a distinct cluster of tumor epithelial cells in EBVaGC, which exhibit elevated proliferative activity and enhanced immunogenicity. These cells overexpress interferon-stimulated genes, including MHC class II molecules (e.g., HLA-DMA, HLA-DPB1, HLA-DRA) and IRF8, which may improve immune recognition. Conversely, tumor suppressors and differentiation markers such as TFF1, TFF2, CDHR5, GKN1, and DPEP1 are downregulated, potentially contributing to increased invasiveness and malignant progression (147). The immunogenic phenotype of EBVaGC epithelial cells provides a rationale for MHC class II–targeted immunotherapies aimed at enhancing antitumor immunity (**Figure 3**).

### 4.5 Potential targets

Recent integrative profiling of EBV-associated gastric cancer (EBVaGC) has uncovered a repertoire of actionable targets across three interconnected layers. At the viral level, the nuclear non-coding RNA ebv-circRPMS1 binds and neutralizes p53 (158), miR-BARTs (e.g., BART19-3p) relieve G_2_/M checkpoint control by targeting GADD45B, and secreted EBERs chronically activate TLR3 while up-regulating PD-L1; each step can be precisely intercepted by ASOs, antagomiRs, or TLR3 antagonists (159). Epigenetic–metabolic dependencies are dominated by PIK3CA-activating mutations and 9p24.1 amplification in ~80% of patients, rendering the PI3Kα–mTOR axis essential; EZH2 drives stem-like phenotypes when ARID1A is lost, and virus-induced SREBP1–FASN lipogenic flux sustains membrane biogenesis—alterations already matched to the clinically advanced agents alpelisib (NCT04526470), tazemetostat, and the FASN inhibitor TVB-2640 (154, 155, 160). Within the immune micro-environment, high PD-L1 expression or 9p24.1 amplification predicts marked response to PD-1/PD-L1 blockade, while single-cell sequencing reveals that terminally exhausted CD8^+^ T cells prominently and persistently express LAG-3 after ICI3; the anti-LAG-3 mAb relatlimab plus nivolumab—already approved for melanoma—can be readily translated into basket trials for gastric cancer (148). Additionally, EBV-driven IDO1 over-expression skews tryptophan catabolism toward Treg expansion, positioning IDO1 inhibitors as future partners (156), and the 30% of tumors that abundantly present MHC-II molecules are directly recognisable by CD4^+^ T cells, offering further rationale for MHC-II-enhancing strategies such as 5-aza or CD4 engagers (157).

## 5 Lymphoepithelial carcinoma

### 5.1 Epidemiology and incidence

Lymphoepithelial carcinoma (EBV-positive lymphoepithelial carcinoma, LEC), formerly termed lymphoepithelioma-like carcinoma (LELC), is a malignant neoplasm that morphologically resembles undifferentiated nasopharyngeal carcinoma and is consistently associated with Epstein–Barr virus (EBV). Its defining histopathology is the intimate intermingling of neoplastic epithelial cells with a dense lymphoid infiltrate (161, 162). First linked to EBV in 1985 and systematically confirmed by EBER-ISH in the 1990s (162–164). LEC can arise wherever squamous or glandular epithelium exists, but shows a striking head-and-neck tropism: nasopharynx (~100% EBV-positive), salivary glands (≈100% in South China, Southeast Asia and Inuit populations; ~50% in Western cohorts), sinonasal/oropharyngeal/laryngeal tracts, lung (>90% in Asians), stomach (>80%), and rare esophageal (all reported cases EBV^+^) or hepatobiliary primaries; scattered cases occur in genitourinary organs, breast, skin, thyroid and prostate (165–171). In endemic areas salivary-gland LEC comprises 20–92% of salivary malignancies (annual incidence 2–5/10^5) versus 0.3–0.7% elsewhere (171). Nasopharyngeal LEC, the prototype, yielded an estimated 125 000–143–000 new cases worldwide in 2020 (>95% EBV-related), with Guangdong/Guangxi incidence reaching 25–50/10^5 (4). Prognosis is stage-dependent: salivary-gland LEC 5-year survival ≈75%, advanced disease median 3.1 years (162); nasopharyngeal LEC 70–90% at presentation, recurrence median 7–22 months; pooled thymic LEC (n=58) median 22 months, 5-year survival 34% (164). Distribution mirrors NPC: high rates in South China, Taiwan, Singapore, Malaysia, Vietnam, Philippines, Arctic, parts of North Africa/Middle East, and rising among Asian immigrants in North America and Australia, underscoring pronounced geographic and ethnic clustering (4).

### 5.2 EBV-driven oncogenesis and evasion

In EBV-positive LEC, the virus persists exclusively in a strict viral latency II program, distinguished by stable expression of EBNA1, LMP1, LMP2A/B, and a broad repertoire of BART miRNAs (172). LMP1 constitutively activates NF-κB and PI3K–AKT signaling by mimicking CD40, while LMP2A sustains PI3K–AKT–mTOR signaling. Concurrently, EBNA1 and BART miRNAs contribute to the silencing of key tumor suppressors—including p53, PTEN, and p16(INK4a)—through ubiquitin-mediated degradation or post-transcriptional repression (173, 174). Multi-omic profiling of primary pulmonary LEC by Yang et al. revealed recurrent integration of the viral genome into fragile sites at 1p36, 3p21, 7q11, and 11q23, resulting in focal deletions that disrupt genes such as HLA-DRB1 and SETD2. These EBV-mediated genomic alterations represent a defining molecular signature of LEC (175). To evade immune surveillance, EBV downregulates HLA class I/II molecules, induces PD-L1, IDO1, and IL-10, and suppresses NK-cell–activating ligands, collectively attenuating T-cell and NK-cell cytotoxicity. In parallel, the LMP1/LMP2A–BART axis inhibits mitochondrial apoptosis, necroptosis, and lysosome-dependent cell death while moderately activating autophagy, thereby facilitating malignant transformation and promoting tumor cell survival (175).

### 5.3 Tumor microenvironment in LEC

**Figure 4:**
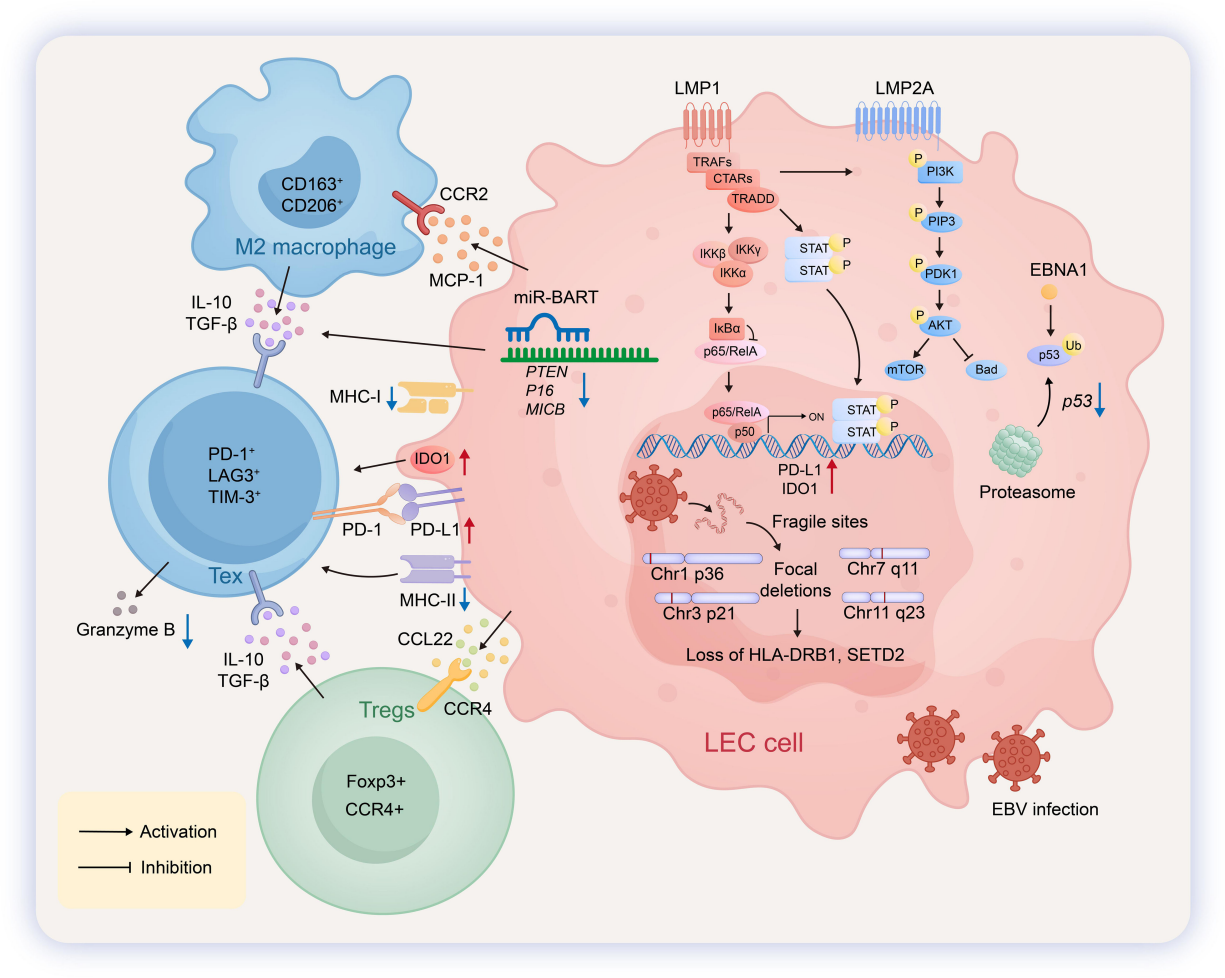
Pathogenic mechanisms and immune microenvironment in LEC. In Lymphoepithelial carcinoma (LEC), EBV establishes a type II latency, expressing EBNA1,LMP1,LMP2A,EBERs. Tumor cells harbor chromosomal fragile sites and focal deletions at regions such as 1p36, 3p21, 7q11, and 11q23, which are associated with the loss of gene expression, including HLA-DRB1 and SETD2. EBV-encoded miR-BARTs mediate the ubiquitination of p16, leading to its subsequent degradation by the proteasome. LMP1 activates the NF-κB pathway to drive the transcriptional upregulation of PD-L1 and IDO1, while LMP2A triggers the phosphorylation cascade of the PI3K/AKT/mTOR pathway. Additionally, EBV engages the JAK/STAT pathway to participate in the transcriptional regulation of PD-L1 and IDO1.Within the tumor microenvironment, M2-polarized macrophages (CD163^+^CD206^+^) are recruited via CCR2 in response to MCP-1 secreted by LEC cells. These macrophages secrete IL-10 and TGF-β to mediate immunosuppression. Exhausted T cells (Tex) interact with LEC cells through the PD-1/PD-L1 axis and are further suppressed by IDO1, IL-10, and TGF-β, resulting in inhibited granzyme B expression and functional exhaustion. Regulatory T cells are recruited via CCR4 upon recognition of CCL22 secreted by LEC cells and contribute to the immunosuppressive milieu by secreting IL-10 and TGF-β.

The immune microenvironment of EBV-positive LEC closely resembles that of nasopharyngeal carcinoma, displaying dense immune infiltration coupled with functional impairment—a phenotype often described as “high-density yet suppressive” (176, 177). CD8^+^ T cells are abundant within and around tumor nests, yet the majority exhibit an exhausted phenotype marked by co-expression of PD-1, TIM-3, and LAG-3, along with low granzyme B expression. Foxp3^+^ Tregs and CD163^+^/CD204^+^ M2 TAMs are preferentially enriched at the invasive margin, where they help establish an immunomodulatory barrier. This immune infiltrate is embedded in a stroma rich in cancer-associated fibroblasts and collagen, which secretes chemokines such as CXCL12 (176–179). A profoundly immunosuppressive cytokine milieu—characterized by elevated levels of IL-10, TGF-β, IDO-1, and PD-L1/PD-L2—drives T-cell exhaustion and enables tumor immune evasion despite robust immune infiltration. Concomitantly, persistent IFN-γ, IL-1β, and TNF-α signaling reflects ongoing virus-associated inflammation. EBV-encoded LMP1/2A and BART microRNAs further reinforce this state by directly upregulating PD-L1 and IDO-1, impairing antigen presentation via NF-κB and JAK/STAT pathways, and promoting Treg recruitment (via CCL22) and M2 macrophage polarization (via MCP-1), thereby sustaining a self-perpetuating immunosuppressive circuit (**Figure 4**) (176, 180–183).

### 5.4 Potential targets

Currently, no targeted therapies are approved specifically for EBV-positive LEC. Off-label use of PI3K–AKT–mTOR inhibitors or personalized regimens guided by next-generation sequencing is sometimes attempted, though their efficacy remains to be formally validated (184). In the immunotherapy domain, PD-1/PD-L1 blockade has shown survival benefits in advanced disease, with retrospective series reporting objective response rates of 30–60%. Higher PD-L1 presentation and a dense infiltration of CD8^+^ T cells are associated with improved responses (185). EBV-specific cytotoxic T lymphocytes (EBV-CTLs) have achieved durable complete remission rates of 60–80% in type II latency tumors, including pulmonary and gastric LELC; however, manufacturing delays and the lack of prospective clinical data limit their widespread application (184). Histone deacetylase inhibitors (e.g., anatinostat) combined with ganciclovir, which exploit viral lytic induction, have yielded clinical responses in approximately 40% of cases (78). In addition, multiple clinical trials are actively evaluating immune checkpoint inhibitors in combination with chemotherapy, targeted agents, or radiotherapy (186, 187).

## 6 Diffuse large B cell lymphoma

### 6.1 Epidemiology and incidence

Diffuse large B-cell lymphoma (DLBCL) is the most prevalent form of non-Hodgkin lymphoma (NHL), comprising between a quarter and one-half of all NHL diagnoses. This aggressive cancer originates from mature B cells. This malignancy is marked by brisk proliferation rates and exhibits substantial variability in its clinical presentation and disease course (188). Research by Oyama et al. in 2007 brought to light the initial evidence connecting Epstein–Barr virus (EBV) to DLBCL (189), and subsequent global studies have revealed substantial geographic variation in the proportion of EBV-positive cases among all DLBCL: 11.4% in Japan, 4.5% in Taiwan, 14.0% in Peru, 5.3% in Turkey, 2.5% in Europe, and 4.0% in Western countries overall (190). These variations likely reflect differences in geography, ethnicity, and immune status. EBV-positive DLBCL typically occurs in older patients compared to EBV-negative cases and is more prevalent in the elderly (190, 191). Although EBV-positive DLBCL is relatively uncommon, it is associated with high mortality and poorer prognosis than its EBV-negative counterpart. Studies report a 2-year overall survival of 40% ± 10% and a 2-year progression-free survival of 36% ± 9% in EBV-positive patients (191, 192). The incidence is relatively higher in East Asia (e.g., Japan and South Korea) and Peru, and lower in Western countries, with a slight male predominance (190). These epidemiological features provide a critical foundation for further investigation into the pathogenesis and clinical management of EBV-positive DLBCL.

### 6.2 EBV-driven oncogenesis

The pathogenesis of EBV-positive DLBCL is multifactorial, involving complex virus–host interactions. Following infection, EBV establishes lifelong latent infection in B cells, primarily adopting latency type III, and expresses viral antigens including EBNA1, EBNA-LP, EBNA3 proteins, EBERs, LMP1, and LMP2A/B (190, 193). Among these, LMP1 mimics CD40 receptor activation, recruits TRAFs, and constitutively activates NF-κB and PI3K/Akt signaling, thereby promoting B-cell proliferation, survival, and inhibition of apoptosis (17, 194, 195). LMP2A mimics B-cell receptor signaling, driving B-cell activation and proliferation while modulating the immune microenvironment (196). Genetically, EBV-positive DLBCL is associated with activation of the JAK-STAT pathway and mutations or amplifications in oncogenes such as NOTCH1/2 and PIM1 (197). Tumor suppressor genes including CD58, B2M, and TP53 are frequently mutated, facilitating immune evasion and tumor survival (198). Chromosomal alterations are also common, such as amplifications of 1q24.3 and 9p24.1, leading to FASL and PD-L1 overexpression, which respectively influence apoptosis and immune escape (198, 199).

The risk of developing EBV-positive DLBCL escalates with age, where immunosenescence is a key contributing factor to this increased susceptibility. Immunosenescence drives a decline in lymphocyte diversity and inflammatory homeostasis, culminating in a pro-tumorigenic, chronically inflamed microenvironment (189, 189, 200, 201). Collectively, the pathogenesis of EBV-positive DLBCL arises from the interplay of EBV infection, viral protein functions, immune dysregulation, genetic alterations, and aberrant activation of cellular signaling pathways.

### 6.3 HLA and EBV immune evasion

Immune escape in Epstein–Barr virus-positive diffuse large B-cell lymphoma (EBV^+^ DLBCL) is orchestrated by the combined effects of host HLA genetics and viral molecules. First, low-presenting HLA-I alleles (A02:01, B08:01, C07:01) and the DRB115:01–DQB1*06:02 haplotype predispose individuals to symptomatic primary EBV infection and high latent viral loads; these alleles remain enriched in subsequent lymphoma tissue, indicating persistently inefficient presentation of EBV epitopes to CD8^+^ T cells and underscoring that suboptimal antigen presentation spans the entire latency-to-malignancy trajectory (198, 202). Second, the viral protein EBNA2 competes with CIITA for binding to MHC-II enhancers, repressing HLA-II transcription; RNA-seq shows markedly reduced CIITA mRNA in EBV^+^ DLBCL, with only ~30% of cases retaining HLA-II expression (203). LMP1 and LMP2A up-regulate PD-L1 via the NF-κB–JAK/STAT axis, and LMP2A further amplifies inhibitory signalling by phosphorylating SYK (204), while miR-BARTs down-regulate the PBRM1–FOXP1–PIAS3 network, cooperatively impairing antigen processing and enhancing STAT3-driven PD-L1 expression (203). Additionally, inactivating B2M mutations (≈11%) cause complete loss of HLA-I, CD58 mutations (≈11%) disrupt CD2–CD58 co-stimulation, and 9p24.1 amplification (≈20%) leads to PD-L1 over-expression; together with virus-induced PD-L1, these alterations create a profoundly immunosuppressive microenvironment that enables dual immune escape (198, 205). Clinically, patients with low or mutated HLA-I/II may derive limited benefit from single-agent PD-1/PD-L1 blockade; combination with JAK2 inhibitors, HDAC inhibitors, or CD58–CD2 agonists is therefore worth exploring. In young individuals with sustained high EBV loads who carry susceptible HLA genotypes, prophylactic EBV-CTL infusion could potentially reduce lymphoma risk.

### 6.4 Tumor microenvironment

The EBV^+^DLBCL microenvironment features a high degree of M2 macrophage polarization, evidenced by an elevated CD163/CD68 ratio that independently predicts advanced stage and poor prognosis (206). Single-cell and spatial transcriptomics reveal that the viral latent membrane protein LMP1 up-regulates CSF1 and IL-10 in tumor cells via the NF-κB/STAT3 axis, while LMP2A enhances CCL5 secretion; together these factors recruit peripheral monocytes and drive their differentiation into the CD163^+^CD206^+^ M2 phenotype. After differentiation, TAMs autocrinely produce IL-10 to establish a positive feedback loop and highly express MMP9 to degrade extracellular matrix and release TGF-β, further suppressing NK and CD8^+^ T cells and thereby constructing an “EBV → M2 → immune exhaustion” cascade (111, 207). In addition, dendritic cells (DCs) residing in the immune microenvironment of EBV-positive DLBCL mostly co-express IDO1 (the rate-limiting enzyme of tryptophan metabolism), thereby promoting tumor immune escape (13).

**Figure 5:**
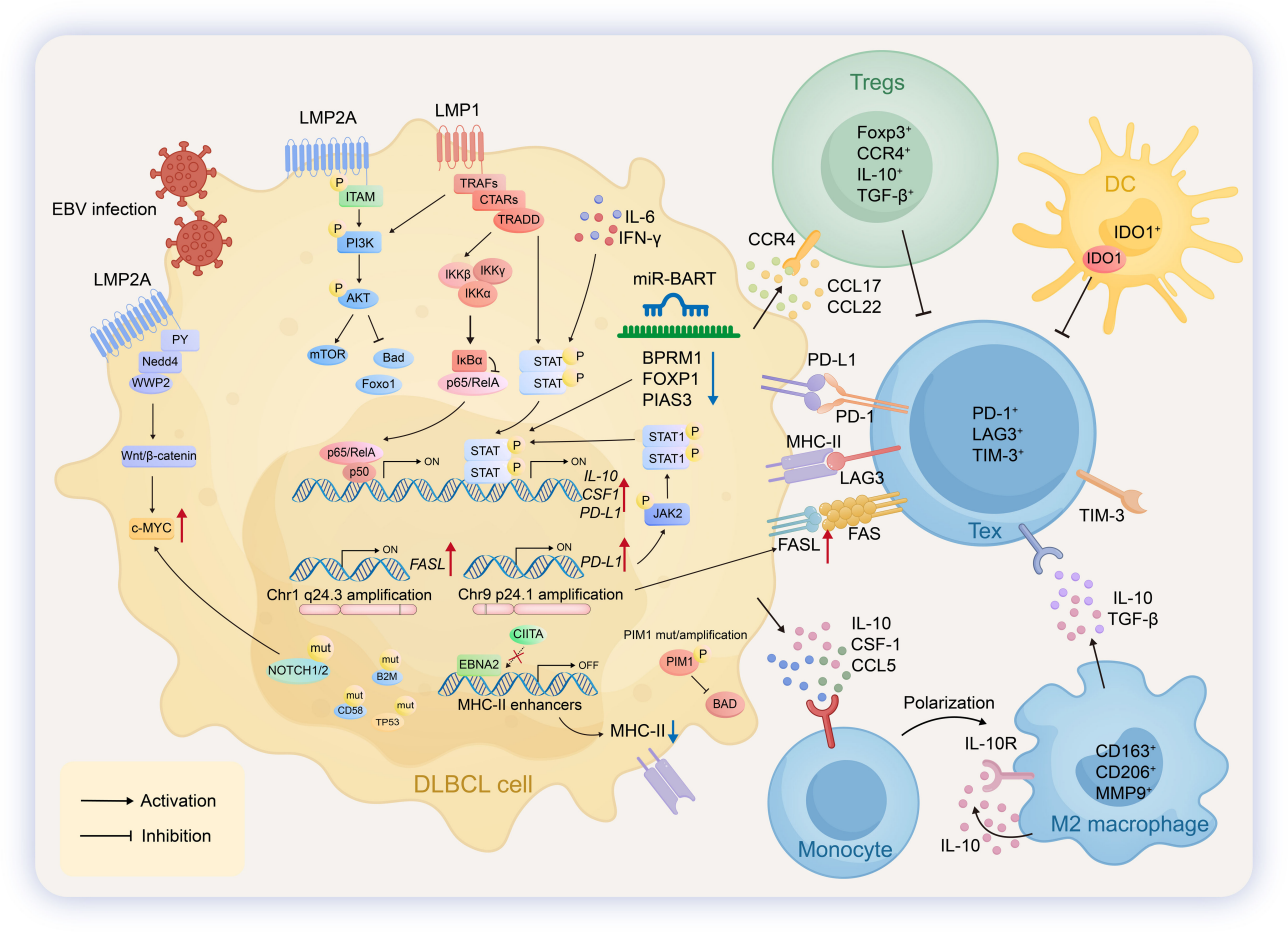
Pathogenic mechanisms and immune microenvironment in DLBCL. In Hodgkin lymphoma (HL), EBV establishes a type II latency. The viral latent membrane protein LMP1 activates the NF-κB pathway to upregulate PD-L1 and PD-L2, while LMP2A triggers a phosphorylation cascade via the PI3K/AKT pathway. Viral non-coding RNAs also contribute: miR-BART2-5p downregulates MICB expression, thereby reducing NK cell cytotoxicity, and the EBERs activate macrophage-associated signaling through the TLR3 pathway. Other EBV-encoded miRNAs participate in the transcriptional regulation of cellular gene expression. The accompanying inset figure illustrates additional key mechanisms that impair CD8^+^ T cell recognition, including the inhibition of EBNA1 antigen processing by its Gly-Ala repeat domain, as well as the blockade of TAP-mediated peptide transport by the lytic protein BNLF2a and the accelerated endocytosis of MHC-I molecules by BILF1.NK cells recognize tumor cells via the NKG2D-MICB axis to exert cytotoxicity. CD8^+^ T cells mediate cytotoxic effects through TCR recognition of tumor cell MHC-I. A subset of naïve-like B cells expresses CXCR5 and secretes Galectin-9, which binds to TIM-3. A distinct population of immunosuppressive CD4^+^ T cells (LAG3^+^TIM-3^+^ Tr1-like) engages multiple inhibitory checkpoints: they interact with PD-L1 on tumor cells via PD-1, with MHC-II via LAG3, and with Galectin-9 from naïve-like B cells via TIM-3, while secreting IL-10 and TGF-β. Macrophages are recruited and polarized toward an M2 phenotype via the CCR2-MCP-1 axis. Their function is further modulated by the EBERs/TLR3 pathway, leading to the secretion of IL-10 and TGF-β, which reinforces the immunosuppressive milieu.

In EBV-positive settings, an increase in CD4^+^ T cells is concurrently observed with a notably potent immunosuppressive function of regulatory T cells (Tregs), principally via IL-10 production (13, 208). Within the EBV^+^ tumor milieu, incipient cytotoxic and virus-specific T-cell responses are overridden by a dominant immunosuppressive network. This network, composed of checkpoint signaling, suppressive cell subsets, and inhibitory soluble factors, culminates in T-cell exhaustion and loss of immune surveillance (13). The microenvironment also features elevated levels of inhibitory cytokines including IL-10 (13), and upregulation of immune checkpoint molecules including PD-L1, PD-L2, LAG3, and TIM3, which collectively induce T-cell exhaustion (13, 209–211). Overall, the immune microenvironment in EBV-positive DLBCL is markedly immunosuppressive and immune-tolerant (**Figure 5**) (13, 212).

### 6.5 Potential targets

The treatment landscape for EBV-positive DLBCL is rapidly evolving, with growing interest in immunotherapeutic and targeted approaches. Immune checkpoint inhibitors targeting the PD-1/PD-L1 axis, such as pembrolizumab, have shown promise either as monotherapy or in combination with R-CHOP, improving outcomes in a subset of patients (213, 214). EBV-directed therapies are also under investigation, including LMP1-targeted CAR-T cells, LMP1-based mRNA vaccines, and strategies targeting EBNA1 (215, 216). The role of BTK inhibitors such as ibrutinib remains controversial in specific age groups, and proteasome inhibitors (e.g., bortezomib) as well as PI3K and mTOR inhibitors are being explored, though clinical outcomes are not yet clearly established (215, 217). In the realm of cell therapy, a case reported by Min Yu et al. demonstrated successful treatment of refractory retroperitoneal EBV-positive DLBCL with secondary hemophagocytic lymphohistiocytosis using sequential PD-1 inhibition and CAR-T cell therapy (218).

In summary, while several emerging therapeutic strategies show potential, they remain under development and require further validation. Future clinical trials and translational studies are essential to optimize treatment efficacy and improve survival outcomes for patients with EBV-positive DLBCL.

## 7 NKT-cell lymphoma

### 7.1 Epidemiology and incidence

NKTCL is defined as a highly aggressive, rare non-Hodgkin lymphoma primarily of natural killer cell origin, and less commonly, of cytotoxic T-lymphocytes. While mucosal involvement is common, its pathological distinction places it outside the conventional mucosa-associated lymphoid tissue (MALT) spectrum in current classifications. During the 1990s, the causative role of Epstein-Barr virus (EBV) in extranodal NK/T-cell lymphoma (ENKTL) was definitively recognized by the scientific community (219). In 1994, the World Health Organization (WHO) formally recognized EBV infection as a defining characteristic of this entity in its lymphoma classification (220). According to the 2016 WHO classification of hematopoietic and lymphoid tumors, EBV positivity is now an essential diagnostic criterion for NKTCL (221).

NKTCL constitutes less than 2% of all T-cell lymphomas and demonstrates a striking geographic distribution, being most prevalent in East Asia and Latin America, while remaining rare in Europe and North America (222). In regions such as China, Japan, South Korea, and Hong Kong, the incidence is notably elevated. Southeast Asia reports an incidence of approximately 0.25 per 100, 000, compared to less than 0.1% in Western populations (223). NKTCL follows an aggressive clinical course with generally poor outcomes (224). Although therapeutic advances have gradually improved survival in recent years, overall mortality remains high. The 5-year survival rate for nasal-type NKTCL is approximately 54%, while non-nasal forms exhibit a significantly poorer prognosis, with a 5-year survival of only 34% (225, 226).

### 7.2 Pathogenesis

The pathogenesis of NKTCL involves a complex interplay of viral, genetic, and microenvironmental factors. Frequent deletions at chromosome 6q21 lead to the silencing of tumor suppressor genes including PRDM1, ATG5, and AIM1 (227–230). Concurrently, TP53 inactivation and aberrant activation of oncogenes including PD-L1 and MYC constitute central oncogenic drivers (227). Recently, Lu Jiang et al. demonstrated that DDX3X mutations impair RNA helicase activity, thereby accelerating NK-cell cycle progression and activating NF-κB and MAPK signaling, ultimately contributing to poor prognosis (231).

### 7.3 EBV-driven oncogenesis

Epstein-Barr virus (EBV) is central to the pathogenesis of natural killer/T-cell lymphoma (NKTCL), wherein it maintains a predominant type II latency program characterized by the expression of key latent proteins, including EBNA1, LMP1, and LMP2 (10, 232). These latent proteins exert multiple oncogenic effects: LMP1 activates NF-κB and PI3K/Akt signaling to upregulate survivin and inhibit apoptosis, while also inducing PD-L1 and IL-2Rα to facilitate immune evasion and promote tumor proliferation (233–236). Additionally, EBV skews T-cell differentiation toward a Th2-like response via upregulation of GATA3 and suppression of T-bet, further enabling immune escape (219).

Molecular subtyping has revealed coordinated heterogeneity between EBV gene expression and host genomic alterations. The TSIM, MB, and HEA subtypes exhibit distinct EBV latency patterns: the MB subtype shows type I latency with LMP1 suppression; HEA and TSIM display type II latency, with HEA characterized by high BNRF1 expression driving viral replication, and TSIM showing elevated BALF3, potentially inducing genomic instability (227). These viral patterns align with host molecular features—MYC activation in MB suppresses viral antigen expression, aberrant histone acetylation in HEA promotes lytic reactivation, and JAK-STAT activation in TSIM fosters an immune-evasive microenvironment (227).

EBV also modulates cell death pathways to promote survival. LMP1 upregulates Bcl-2 and downregulates Bax via NF-κB, inhibiting apoptosis. EBNA1 enhances survivin expression through STAT1 activation and TGF-β1 suppression (233, 237). EBV-mediated PI3K/Akt activation and p53 pathway interference further reinforce apoptosis resistance (233). While direct evidence is limited, EBV may also influence pyroptosis and necroptosis through inflammatory modulation (238, 239). Autophagy exhibits dual roles—some EBV proteins induce pro-survival autophagy, whereas excessive activation may trigger cell death; the precise mechanisms require further investigation (239).

### 7.4 HLA and EBV immune evasion

In Epstein–Barr virus-positive (EBV^+^) extranodal NK/T-cell lymphoma (NKTCL), human leukocyte antigen (HLA) molecules shape immune surveillance and tumour immune escape by modulating the efficiency of antigen presentation. On the one hand, c-Met—identified as a novel tumour-associated antigen—is broadly expressed by EBV^+^ NKTCL cells, and its ligand hepatocyte growth factor (HGF) is secreted autocrinally to establish an HGF/c-Met loop that drives proliferation. Three c-Met-derived CD4^+^ T-cell epitopes (c-Met_278_–_292_, _817_–_831_ and_1244_–_1258_) have been characterised; they are presented by HLA-DR molecules (restricted to HLA-DR9, DR12 and DR53, respectively) and elicit specific helper-T-cell (HTL) responses. These HTLs not only recognise and kill HLA-DR-matched, c-Met^+^ NKTCL cells directly, but also respond to tumour antigens cross-presented by dendritic cells, thereby amplifying anti-tumour immunity (240). Moreover, c-Met inhibitors such as ARQ197 reduce tumour-cell TGF-β secretion, relieve immunosuppression, and further enhance HTL recognition, establishing c-Met as a candidate target for HLA-DR-restricted immunotherapy in EBV^+^ NKTCL (240, 241).

Alternatively, genome-wide association studies (GWAS) have found that the inherited risk of EBV^+^ NKTCL is intimately linked to functional polymorphisms within HLA-DP and HLA-DR molecules. A haplotype encoding Gly_84_-Gly_85_-Pro_86_-Met_87_ at the rim of the HLA-DPB1 peptide-binding groove markedly increases NKTCL risk (OR = 2.38), probably by altering peptide-binding conformation and reducing the presentation of EBV antigens (223). Epidemiological data further reveal that the frequency of the HLA-A02:01 allele is significantly lower in NKTCL patients than in controls, implying that HLA-A02:01-restricted cytotoxic T lymphocytes may confer protection against EBV-LMP1-expressing tumour cells; loss of this allele may facilitate immune escape (242).

A subsequent large-scale GWAS across multiple East-Asian populations identified HLA-DRB1 and IL18RAP as additional, independent susceptibility loci for EBV^+^ NKTCL. The IL18RAP risk allele up-regulates its own expression, accelerates cell-cycle progression, and promotes NKTCL proliferation, whereas the HLA-DRB1 risk variant—located in peptide-binding pocket 7 (47Y-67L)—may impair presentation of EBV antigens and attenuate CD4^+^ T-cell surveillance, fostering lymphomagenesis. Individuals carrying risk alleles at all three loci (HLA-DPB1, HLA-DRB1, and IL18RAP) exhibit an >18-fold increase in disease risk (223). Collectively, these findings underscore the pivotal role of both HLA class I and class II molecules in EBV immune escape within NKTCL, indicate that defective antigen presentation constitutes a central mechanism underlying EBV evasion, and provide a theoretical framework for immunotherapeutic and precision-prevention strategies against NKTCL.

On this basis, the virus itself further reinforces the escape barrier through “immune stealth” and “immune suppression”. EBV almost universally persists in Latency II, expressing only EBNA1 and LMP1/2A, thereby preserving its oncogenic potential while minimizing CTL targets. Among these, LMP1 markedly up-regulates tumour-cell PD-L1 via activation of the MAPK/NF-κB axis, directly suppressing PD-1^+^ CTLs, and represents the best-documented, NKTCL-specific immune-evasion pathway (243–245). At the population level, the low frequency of HLA-A*02:01 creates a “genetic bottleneck”, indicating that carriers efficiently eliminate LMP1^+^ pre-malignant clones and thus indirectly select for immune-escape variants (242). Although EBV-miR-BART has been shown in NPC to down-regulate the NK-activating ligand MICB (112), its high expression in NKTCL remains functionally unvalidated, yet is presumed to cooperate in impairing NK-cell surveillance. Collectively, HLA genetic polymorphisms and the EBV transcriptional programme act in concert to shape the antigen-presentation defects and immunosuppressive microenvironment characteristic of NKTCL, providing a rationale for combined PD-1/PD-L1 blockade with HLA-restricted personalised vaccines or LMP1/BART-targeted interventions.

### 7.5 Tumor microenvironment

Single-cell RNA sequencing (scRNA-seq) has delineated three immune microenvironment patterns in NKTCL with prognostic significance: the immune-desert subtype, devoid of T cells and myeloid dendritic cells (MDCs), shows high EBV gene expression and suppressed interferon/TLR signaling, correlating with the worst outcomes (5-year PFS 58.7%, OS 43.1%); the immune-excluded subtype, with T-cell infiltration but absent MDCs, exhibits intermediate prognosis (5-year PFS 73.3%, OS 66.4%); and the immune-inflamed subtype, enriched for T cells and MDCs despite partial exhaustion, is associated with the most favorable survival (5-year PFS 83.9%, OS 89.4%) (246).

**Figure 6:**
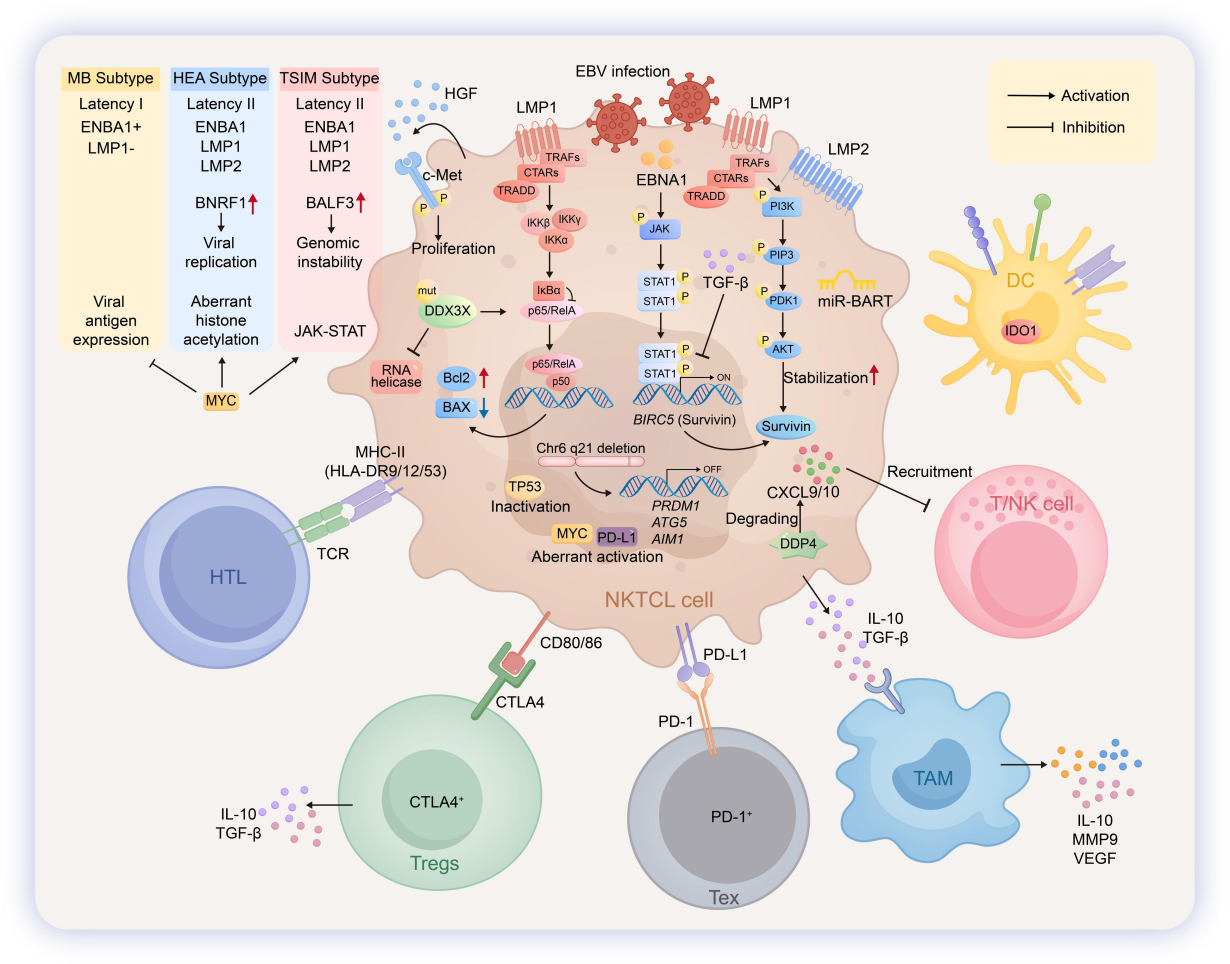
Pathogenic mechanisms and immune microenvironment in NKTCL. In Diffuse large B-cell lymphoma (DLBCL), EBV establishes a type III latency. DLBCL cells harbor recurrent genomic alterations including amplifications at Chr1q24.3 and Chr9p24.1, mutations and/or amplifications of PIM1, and mutations in genes such as NOTCH1/2, CD83, and TP53. Concurrently, aberrant regulation of MHC-II enhancers results in the downregulation of MHC-II expression. LMP1 activates the NF-kB pathway to drive the transcription of IL-10 and PD-L1. LMP2A, through its ITAM motifs, triggers the PI3K/AKT/mTOR phosphorylation cascade to regulate cell survival and proliferation, while also contributing to c-MYC upregulation via the Wnt/bcatenin pathway. The JAK2/STAT1 axis is activated and participates in the transcriptional activation of PD-L1 and IL-10. Furthermore, EBNA2 binds to MHC-II enhancers, leading to their epigenetic silencing. The tumor microenvironment is profoundly immunosuppressive. Regulatory T cells (Tregs) are recruited via the CCR4-CCL17/22 axis and secrete IL-10 and TGF-b. Dendritic cells express IDO1, fostering an inhibitory milieu. Exhausted T cells (Tex) co-express multiple inhibitory receptors such as PD-1, LAG3, and TIM-3; their function is further suppressed by the FAS/FASL pathway and cytokines including IL-10 and TGF-b. Monocytes, under the influence of factors like CSF-1 and CCL5 secreted by DLBCL cells, polarize into M2-type macrophages that reinforce immunosuppression through the secretion of IL-10 and TGF-b.

Malignant NK cells display substantial heterogeneity. The NK_C9_CXCL13 subcluster, expressing LMP1, activates JAK-STAT and NF-κB pathways, driving proliferation and immune evasion through secretory mediators (227, 247). Tumor-infiltrating T cells are often exhausted or regulatory (Tregs), expressing PD-1 and CTLA-4, while tumor-associated macrophages (TAMs) contribute to immunosuppression via cytokine secretion and checkpoint expression (247, 248). Malignant cells secrete DPP4, degrading CXCL9/10 and impairing T/NK-cell recruitment (247). The GPCR pathway—particularly CCR1, highly expressed in EBV-infected cells—emerges as a key mediator of immune suppression and proliferation. Targeting CCR1 in organoid models reduces EBV load and tumor growth while enhancing T-cell activation, highlighting its therapeutic potential (**Figure 6**) (246, 249).

### 7.6 Potential targets

Although conventional chemotherapy has improved outcomes for EBV-positive NKTCL, relapsed/refractory disease remains challenging. Recent advances in immunotherapy and targeted agents have opened new avenues. PD-1 blockade (e.g., pembrolizumab) achieves objective response rates of 44–47% in relapsed/refractory NKTCL, with higher PD-L1 expression correlating with improved response (213, 234, 245, 250–253). CTLA-4 inhibitors are under investigation, supported by their efficacy in other lymphomas (224). Monoclonal antibodies targeting CD30, CD52, and CD38 have also shown activity (254–258). Cellular immunotherapies, including anti-CD30 and anti-CD7 CAR-T cells, as well as EBV-specific cytotoxic T lymphocytes (CTLs), have demonstrated promising efficacy in early studies (255, 259). Immunomodulatory agents such as thalidomide, combined with chemotherapy, have also improved response rates (255).

Despite these advances, challenges including treatment-related toxicities and acquired resistance remain. Future efforts should focus on rational combination strategies and biomarker-driven patient selection to improve outcomes and minimize adverse effects.

## 8 Burkitt lymphoma

### 8.1 Epidemiology and incidence

Burkitt lymphoma (BL) is a very aggressive form of B-cell non-Hodgkin lymphoma (NHL). The worldwide incidence of NHL stands at an estimated 6.0 cases per 100, 000 individuals, based on 2022 figures released by the International Agency for Research on Cancer (IARC) (40). BL typically accounts for 1–2% of all NHL cases, with a relatively low incidence in adults and a predominance in children. A study published in Frontiers in Public Health analyzing global childhood BL (ages 0–14) from 1990 to 2021 reported 4, 083 new cases and 3, 065 deaths in 2021. Both incidence and mortality rates have increased since 1990, particularly in sub-Saharan Africa and among children aged 5–9 years (260). In 1964, Epstein, Achong, and Barr first discovered a novel herpesvirus particle—later named Epstein–Barr virus (EBV)—in cultured BL cells using electron microscopy (1). The disease is categorized into three major epidemiological subtypes: endemic, sporadic, and immunodeficiency-associated Burkitt lymphoma, with EBV prevalence varying considerably among them. Endemic BL, occurring primarily within malaria hotspots, including parts of equatorial Africa, shows the strongest EBV association, with >95% of cases being EBV-positive. Sporadic BL, found worldwide and more common in Europe and North America, is EBV-associated in only about 30% of cases. EBV co-infection is detected in a moderate proportion (25–40%) of immunodeficiency-associated BL cases, a category that primarily includes patients with HIV or a history of organ transplantation (261).

### 8.2 Pathogenesis and EBV-driven oncogenesis

Burkitt lymphoma is a prototypical aggressive B-cell malignancy defined by chromosomal translocations involving the MYC oncogene and immunoglobulin loci, which drive uncontrolled cellular proliferation. The majority of cases (approximately 70–80%) carry the t (8,14)(q24;q32) translocation, fusing MYC with the immunoglobulin heavy chain locus. Another 15% and 5% of cases harbor t (2,8)(p12;q24) and t(8;22)(q24;q11), involving the immunoglobulin κ and λ light chain loci, respectively (262, 263). However, MYC activation alone typically induces strong apoptotic signals that impede stable clonal expansion (264). In this context, EBV plays a pivotal role by providing survival support to MYC-translocated cells through its latent gene expression program, immune evasion strategies, and multiple regulatory pathways (265). The EBV latency program in Burkitt lymphoma is remarkably uniform, with virtually all cases adopting Latency I. This state is defined by a hallmark expression profile limited to EBNA1, EBERs, and BART miRNAs. This highly restricted latency pattern maintains viral genome persistence while minimizing immunogenicity, thereby facilitating tumor immune evasion (266–268). Furthermore, structural properties of EBNA1 impair MHC-I–mediated antigen presentation (15, 269), while BART miRNAs target pro-apoptotic and immune-related genes such as BIM, PUMA, and MICB, enhancing cell survival and promoting immune escape (270, 271). Through these mechanisms, EBV effectively counterbalances MYC-induced apoptotic signals. In summary, in MYC-driven BL, EBV’s primary role is not to drive proliferation but to ensure tumor cell survival by resisting apoptosis via restricted latency programs (272).

The cooperation between EBV and MYC is central to BL pathogenesis. EBNA1 and LMP2A enhance MYC-dependent proliferation and metabolic reprogramming through epigenetic modulation and activation of signaling pathways such as PI3K/AKT (273). In addition, EBV-encoded miRNAs modulate the TP53 pathway, attenuating the DNA damage response and promoting genomic instability (274). EBV also interacts with host super-enhancer networks, sustaining high-level expression of MYC and other oncogenes (275).

### 8.3 HLA and EBV immune evasion

HLA genotype critically influences host control of EBV infection. For instance, in the context of Burkitt lymphoma, certain HLA alleles are implicated in protection while others elevate risk. Efficient EBV antigen presentation linked to HLA-A*02 may be protective, in contrast to risk-enhancing alleles like HLA-DQA1*04:01. This is particularly relevant in malaria-endemic regions, where malaria impairs EBV-specific T-cell responses and, in conjunction with certain HLA backgrounds, amplifies EBV’s oncogenic potential (276, 277). In terms of immune evasion, Latency I itself represents a “low-immunogenicity” program. Mechanisms such as translational regulation of EBNA1, immune mediator suppression by EBERs, and chemokine downregulation by BART miRNAs collectively dampen immune surveillance. Moreover, elevated PD-L1 expression has been observed in some BL samples, suggesting that EBV may further exploit the PD-1/PD-L1 axis to suppress T-cell activity and enhance immune escape (278, 279). Regulation of cell death pathways represents another critical mechanism through which EBV supports BL survival. Whereas MYC activation alone induces strong intrinsic apoptotic signaling, EBV counteracts this through EBNA1, BART miRNAs, and viral BCL-2 homologs BHRF1/BALF1, effectively inhibiting apoptosis and promoting tumor cell survival (280, 281).

### 8.4 Tumor microenvironment

Recent single-cell transcriptomic studies have revealed that the non-malignant compartment of BL specimens is dominated by immune cells, with T cells and myeloid cells constituting the most abundant populations. Within the T-cell compartment, two distinct states are observed: a “memory-like” phenotype marked by TCF7, IL7R, and CCR7 expression, and an “exhausted-like” phenotype expressing PDCD1, LAG3, and HAVCR2. Similarly, the myeloid compartment exhibits both inflammatory and regulatory subsets, reflecting considerable heterogeneity. Spatial differences in immune infiltration have also been noted across sampling sites (e.g., lymph nodes versus effusion fluid). Importantly, tumor and microenvironmental features identified at the single-cell level align with functional and biomarker validation in independent cohorts—for example, the association of TPM2 expression with prognosis. Collectively, these findings delineate a tumor ecosystem in which highly proliferative tumor cells are embedded in a microenvironment characterized by concurrent immune activation and pronounced immunosuppression (282).

**Figure 7:**
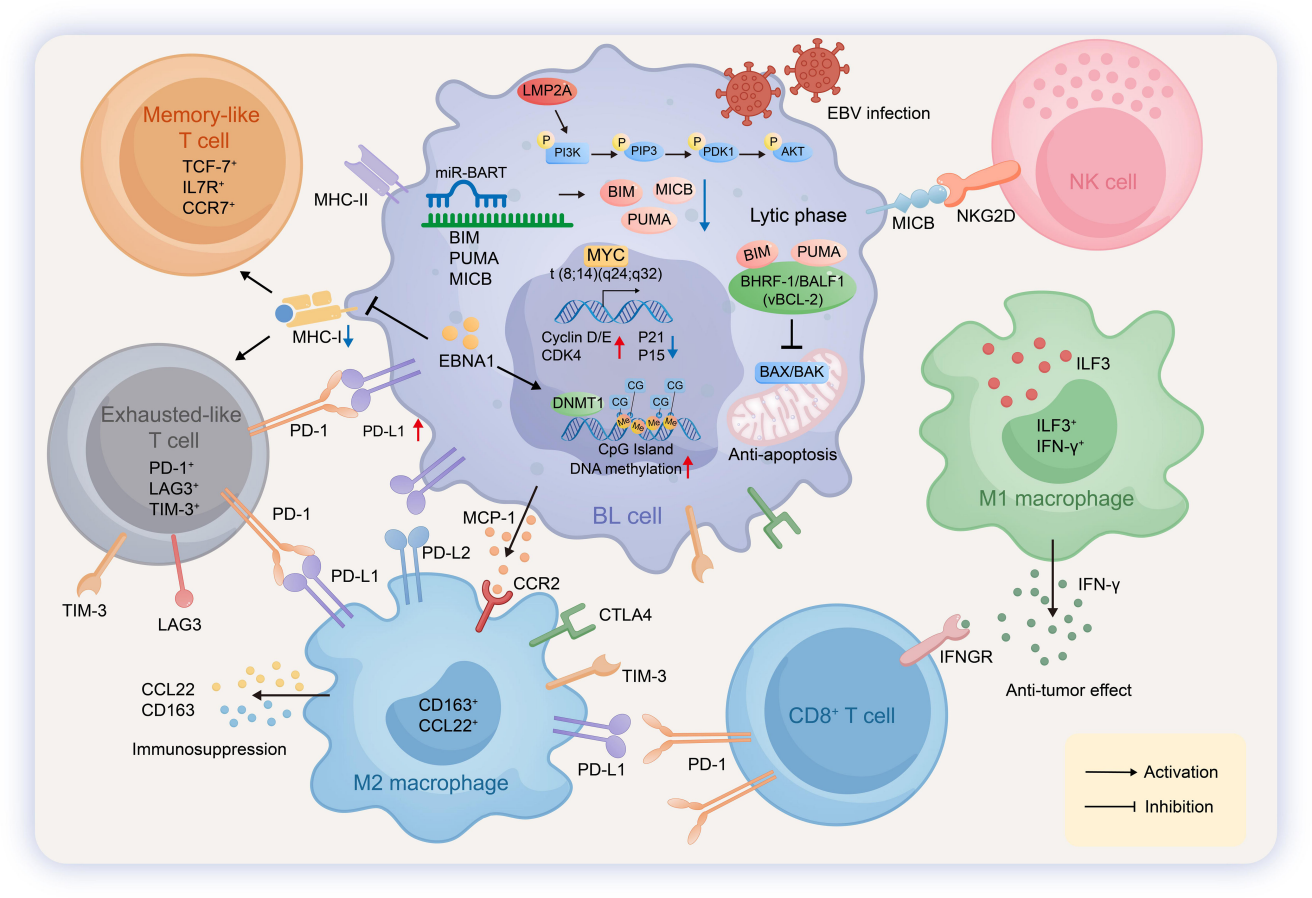
Pathogenic mechanisms and immune microenvironment in BL. In EBV-associated gastric cancer(EBVaGC), EBV establishes a type I/II latency. In EBV-associated gastric carcinoma (EBVaGC), the virus drives tumorigenesis through constitutive activation of multiple oncogenic signaling pathways. The NF-κB axis upregulates anti-apoptotic genes including BCL-2 and Survivin; the Wnt/β-Catenin and PI3K/AKT/mTOR pathways promote epithelial-mesenchymal transition (EMT) and metastasis; and the JAK/STAT cascade induces the expression of DNA methyltransferases DNMT1/3A/3B, leading to DNA hypermethylation and epigenetic silencing of tumor suppressors such as p16. Tumor cells interact with the microenvironment via secreted factors (CCL17, CCL22) and surface molecules (JAG1, DLL1, PD-L1). EBV infection can also activate the intracellular cGAS-STING-IRF3/IFN signaling axis, which modulates OLFM4 expression. Natural killer (NK) cells recognize tumor cells via the NKG2D receptor; IDO1+ dendritic cells (DCs) contribute to immune tolerance; Macrophages are polarized into M1 and M2 subsets and interact with EBVaGC cells and T cells via the CCR2/MCP-1 axis; PD-1+ ISG-15+ CD8+ T cells are predominantly in a precursor exhausted (Tpex) state; PD-1 blockade can promote their differentiation into effector T cells, cytotoxic T cells (GZMB^+^), or tissue-resident memory T cells; Regulatory T cells (Tregs) mediate immunosuppression through CCR4 and the secretion of IL-10/TGF-β; B cells and plasma cells (CD19^+^CD20^+^) participate in immune regulation by secreting IL-10 and IL-6; Cancer-associated fibroblasts (CAFs) further reinforce the immunosuppressive milieu by secreting IL-6 and TGF-β.

Recent studies on the immune microenvironment of Epstein–Barr virus-positive (EBV^+^) Burkitt lymphoma (BL) have uncovered two diametrically opposed immune phenotypes: an “M1/Th1-type (granulomatous)” pattern and an “M2/exhausted-type (starry-sky)” pattern. Siciliano et al. performed NanoString immune-gene expression profiling on seven EBV^+^ BL cases accompanied by a granulomatous reaction, eight EBV^+^ BL cases with classical starry-sky morphology and eight EBV^-^ BL cases. They observed that granulomatous EBV^+^ BL was characterized by prominent M1-polarized macrophages, up-regulation of pro-inflammatory genes such as IRF3 and IFNG, active CD8^+^ T cells expressing IFN-γ, and the formation of an immune—”hot” microenvironment; clinically, these tumours presented at early stages and a subset underwent spontaneous regression. Conversely, classical starry-sky EBV^+^ BL was dominated by M2-polarized macrophages, expressed immunosuppressive molecules including CD163 and CCL22, exhibited elevated immune-checkpoint genes (PDCD1, CTLA4, TIM3), displayed an immune-exhausted profile, and showed an association with advanced disease stages and rapid progression (283). Research has demonstrated that in Burkitt lymphoma, CD8^+^ T cells exhibit high PD-1 expression, with PD-L1 being primarily localized on tumor-associated macrophages (TAMs). Tumor cells themselves are generally PD-L1–negative, with focal positivity observed only in a subset of EBV-positive cases that display an atypical latency pattern (e.g., LMP2A^+^ tumors) (**Figure 7**) (278).

### 8.5 Potential targets

In recent years, ongoing research efforts in EBV^+^ BL have steadily advanced, identifying a number of promising directions for targeted therapy (284). The viral glycoprotein gp350, selectively expressed during the lytic phase of EBV, has emerged as a candidate for CAR-T-cell therapy and has demonstrated potent cytotoxicity *in vitro* and in animal models. M2-polarized tumour-associated macrophages (TAMs) abundantly infiltrate EBV^+^ BL, produce immunosuppressive molecules such as CD163 and CCL22, and can be re-educated toward an M1 phenotype by CSF1R blockade, thereby enhancing anti-tumour immunity. Concurrently, immune-checkpoint proteins including PD-1, CTLA4 and TIM3 are up-regulated in EBV+ BL, indicating that checkpoint inhibitors may hold clinical value (283). In addition, EBV-encoded miRNAs (e.g., miR-BHRF1–2 and miR-BART6-3p) modulate host genes to facilitate immune escape and promote tumour-cell survival; antisense oligonucleotides or miRNA replacement strategies are therefore being explored as novel therapeutic tools (278).Epigenetic regulators such as CREBBP and EP300 are over-expressed in the “starry-sky” variant of EBV^+^ BL and may contribute to immune tolerance, while HDAC or BET inhibitors could potentiate the efficacy of immunotherapy (285). Overall, the data demonstrate that EBV^+^ BL harbours a broad spectrum of actionable targets; future treatment paradigms should consider rational combinations that simultaneously engage viral, immune and epigenetic vulnerabilities to overcome current therapeutic limitations.

## 9 Hodgkin lymphoma

### 9.1 Epidemiology and incidence

Between 1987 and 1989, Studies analyzing Hodgkin/Reed–Sternberg (HRS) cells in Hodgkin lymphoma have revealed the presence of both EBV DNA and latent gene expression, establishing the pathological link between HL and EBV (286). The global burden of EBV-associated classical HL (cHL) shows significant geographic variation: recent estimates indicate that EBV-positive cHL accounts for approximately 74% of cases in Africa, 60% in Latin America, 56% in Asia, 36% in Europe, 32% in North America, and 29% in Oceania. Overall, Estimates indicate that EBV is present in about a third of all cHL cases worldwide. The infection burden disproportionately affects younger and older age groups, as well as geographic areas with developing economies (4). According to GLOBOCAN 2022 estimates, HL caused approximately 82, 469 new cases and 22, 733 deaths globally in 2022, accounting for about 0.4% of all new cancer cases and 0.2% of cancer-related mortality (40). Incidence is relatively higher in regions with a high Human Development Index (e.g., Europe and North America), while mortality disproportionately affects low-income countries, reflecting disparities in diagnosis, treatment access, and healthcare infrastructure. The characteristic bimodal age distribution of HL is more pronounced in high-income countries (287).

### 9.2 EBV-driven oncogenesis

In classical Hodgkin lymphoma, Latency type II in EBV is defined by the expression of EBNA1, LMP1, LMP2A/2B, and EBERs, alongside the general absence of EBNA2 (288). These latent gene products provide tumor cells with sustained NF-κB and BCR-like survival signals, along with potent immunomodulatory effects (289–291). In EBV^+^ cHL, viral latent proteins are sufficient to maintain survival and immune-regulatory signaling: LMP1 acts as a constitutively active CD40 mimic, driving NF-κB activation via the TRAF/IKK axis (286); LMP2A delivers a tonic BCR-like survival signal through the PI3K/AKT pathway, compensating for defective BCR signaling in HRS cells (196); and LMP1 expression, together with EBV positivity, is associated with PD-L1 upregulation, supporting activation of the PD-1/PD-L1 immune checkpoint axis (292). Additionally, EBERs enhance HRS cell survival and apoptosis resistance while modulating the microenvironment via IL-10 induction and exosomal TLR3/RIG-I signaling (293). Together, these mechanisms reinforce a phenotype of enhanced survival and profound immunosuppression. HLA class I alleles play a central role in EBV^+^ cHL susceptibility, with contributions from class II. HLA-A 01:01 and HLA-B37:01 are associated with increased risk, whereas HLA-A02:01, HLA-DRB1 15:01, and HLA-DPB101:01 confer protection (294, 295).

### 9.3 HLA and EBV immune evasion

Population-specific variations also exist—for example, HLA-A02:07 in northern Chinese populations has been linked to EBV subtype–specific susceptibility. Overall, HLA-dependent antigen presentation efficiency defines a distinct risk profile for EBV^+^ cHL, differentiating it from EBV^-^ disease. Immune evasion in EBV^+^ cHL is mediated not only by canonical mechanisms-LMP1-driven PD-L1 upregulation, LMP2A-mediated “BCR-like” tonic signaling, and EBER-induced immune remodeling-but also through three additional interconnected pathways. First, antigen presentation is suppressed at multiple levels: the Gly-Ala repeat domain of EBNA1 impairs proteasomal processing and MHC-I loading of its peptides, while the lytic proteins BNLF2a and BILF1 inhibit TAP transport and accelerate MHC-I endocytosis, respectively, limiting CD8^+^ T-cell recognition (296). In addition, EBV glycoprotein gp42 acts as a dual-function molecule: it mediates viral entry into B cells via binding to HLA class II, and in its soluble form (s-gp42), it concurrently promotes immune escape by sterically blocking TCR engagement with MHC-II, thus suppressing CD4^+^ T-cell surveillance (297). Second, viral miRNAs play a coordinated role: EBV-encoded miRNAs suppress IL-12 production, MHC-II transcription, and lysosomal proteases, while miR-BART2-5p specifically targets MICB, attenuating NKG2D-mediated NK and T-cell cytotoxicity (298). Third, microenvironmental remodeling is driven by LMP1, which induces CCL17/CCL22 expression via the NF-κB/ATF2 pathway, recruiting CCR4^+^ Tregs and establishing a suppressive barrier that spatially excludes effector T cells (299, 300) In terms of cell death regulation, EBV latent proteins LMP1 and LMP2A upregulate anti-apoptotic molecules such as BCL2A1 and BCL-XL through NF-κB and PI3K–AKT signaling, reducing apoptotic sensitivity. LMP1 also modulates key necroptosis mediators including RIPK1/RIPK3, further elevating the survival threshold of HRS cells (301, 302).

### 9.4 Tumor microenvironment

A large-scale single-cell RNA sequencing (scRNA-seq) study of adult cHL samples identified a signature subset of LAG3^+^, Tr1-like CD4^+^ T cells that were recurrent across cases and closely associated with the cHL microenvironment. This population produced IL-10 and TGF-β, exhibited immunosuppressive function, and was largely distinct from FOXP3^+^ Tregs. Interestingly, PD-1 expression was primarily found on non-Treg CD4^+^ T cells rather than CD8^+^ T cells. Multiplex imaging showed that LAG3^+^ CD4^+^ T cells were positioned near tumor cells, especially MHC-II–negative HRS cells. Consequently, blocking or depleting these cells ex vivo led to a partial recovery of T-cell activation (303). A complementary scRNA-seq study in pediatric cHL, validated by RNAscope, confirmed enrichment of LAG3^+^/CD69^+^ T cells surrounding HRS cells, especially in HLA-II–deficient contexts. This work also highlighted galectin-1 (LGALS1)–CD69 and HLA-II–LAG3 as reproducible inhibitory interactions and identified potential HRS surface targets such as NRXN3 and LRP8, emphasizing that inter- and intra-tumoral heterogeneity may affect the robustness of therapeutic targets (304). Another single-cell comparative study described a characteristic immune landscape: although the global inflammatory milieu resembled reactive lymph nodes, it coexisted with widespread T-cell dysfunction. IFN-γ pathway activity was globally reduced, while inhibitory checkpoint interactions involving CTLA-4, TIM-3, and LAG-3 were the most prominent and consistent across cases (305).

To bridge single-cell tumor features with peripheral immune indicators, a scRNA-seq study of peripheral blood from the CheckMate 205 trial (evaluating nivolumab in relapsed/refractory cHL) provided key insights. Before PD-1 blockade, CD4^+^ naïve and central memory T cells in patients exhibited reduced TCR clonal diversity, and higher pre-treatment CD4^+^ TCR diversity correlated with improved clinical response. During treatment, two T-cell populations expanded: helper and interferon-activated cytotoxic T cells, and proliferative T cells expressing inhibitory checkpoints such as CTLA-4 and LAG-3. B-cell frequency and BCR diversity declined overall, yet responders showed higher baseline BCR diversity and B-cell counts. Resistance was strongly associated with an expanded population of inflammatory IL-1B^+^ monocytes in blood, which also expressed PD-L1 and SIRPα; phenotypically similar macrophages were identified in tumor tissues. These findings suggest that CD4^+^ T-cell diversity, B-cell response strength, and myeloid-mediated suppression jointly shape patient responses to PD-1 therapy (306).

Analysis of paired diagnostic and relapse samples via scRNA-seq revealed an early-relapse increase in CCR6^+^CXCR5^+^LGALS9^+^ naïve-like B cells, accompanied by a relative decline in memory B cells. This B-cell subset may foster immunosuppression via the galectin-9–TIM-3 axis and HLA-II–dependent interactions with LAG3^+^ CD4^+^ T cells, a pattern corroborated by IHC/IMC validation. These results suggest that targeting the CCR6/CXCR5 migration axis or the TIM-3/galectin-9 checkpoint may remodel the TME and delay early relapse (307). Concurrently, integrated single-cell and spatial imaging analyses of myeloid cells, B cells, macrophages, and HRS cells underscore substantial inter- and intra-tumoral heterogeneity. A practical therapeutic strategy may therefore prioritize targeting HRS surface molecules with stable cross-sample expression (e.g., NRXN3, LRP8) as broadly applicable targets. In contrast, cell–cell communication–dependent pathways (e.g., LAG3, TIM-3, CCR4–CCL22, CXCL13–CXCR5) may be better suited for biomarker-guided or combination approaches (304).

**Figure 8:**
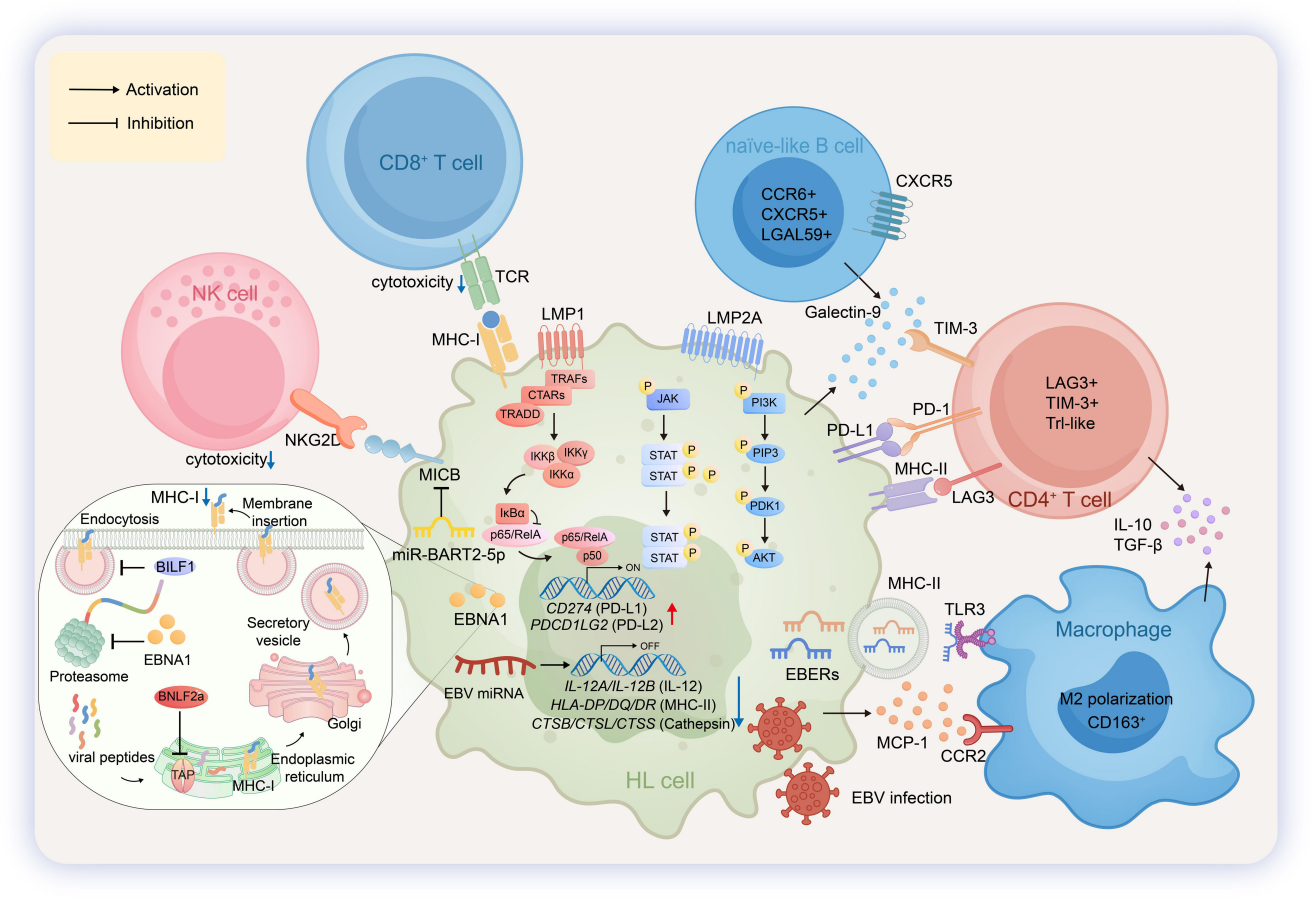
Pathogenic mechanisms and immune microenvironment in HL. In Diffuse large B-cell lymphoma (DLBCL), EBV establishes a type III latency. DLBCL cells harbor recurrent genomic alterations including amplifications at Chr1q24.3 and Chr9p24.1, mutations and/or amplifications of PIM1, and mutations in genes such as NOTCH1/2, CD83, and TP53. Concurrently, aberrant regulation of MHC-II enhancers results in the downregulation of MHC-II expression. LMP1 activates the NF-κB pathway to drive the transcription of IL-10 and PD-L1. LMP2A, through its ITAM motifs, triggers the PI3K/AKT/mTOR phosphorylation cascade to regulate cell survival and proliferation, while also contributing to c-MYC upregulation via the Wnt/β-catenin pathway. The JAK2/STAT1 axis is activated and participates in the transcriptional activation of PD-L1 and IL-10. Furthermore, EBNA2 binds to MHC-II enhancers, leading to their epigenetic silencing. The tumor microenvironment is profoundly immunosuppressive. Regulatory T cells (Tregs) are recruited via the CCR4-CCL17/22 axis and secrete IL-10 and TGF-β. Dendritic cells express IDO1, fostering an inhibitory milieu. Exhausted T cells (Tex) co-express multiple inhibitory receptors such as PD-1, LAG3, and TIM-3; their function is further suppressed by the FAS/FASL pathway and cytokines including IL-10 and TGF-β. Monocytes, under the influence of factors like CSF-1 and CCL5 secreted by DLBCL cells, polarize into M2-type macrophages that reinforce immunosuppression through the secretion of IL-10 and TGF-β.

In summary, The immune landscape of cHL is primarily composed of T cells. These lymphocytes exhibit a suppressed phenotype and are primarily found encircling HRS cells that lack competent antigen presentation, with considerable variability across patients and lesions. Peripheral T- and B-cell clonal diversity correlates with treatment outcome, while inflammatory myeloid cells are enriched in resistant patients. At relapse, suppressive T–B cell interactions become more pronounced(**Figure 8**).

### 9.5 Potential targets

Potential therapeutic strategies for EBV-positive Hodgkin lymphoma (HL) focus on key viral latency proteins, such as LMP1, LMP2A, and EBNA1, along with the host cellular pathways they dysregulate. LMP1 drives proliferation and impairs apoptosis via NF-κB and PI3K–Akt signaling; LMP2A mimics B-cell receptor cues to sustain survival; EBNA1 maintains the viral episome. Exploiting these proteins, strategies under development comprise LMP1/2A-directed CAR-T or TCR-T cells and EBNA1-based vaccines. Host pathways such as NF-κB and PI3K–Akt–mTOR are additionally targetable; corresponding inhibitors (e.g., bortezomib, MK2206) are already in clinical trials. The PD-1/PD-L1 axis represents another critical target, with pembrolizumab demonstrating notable efficacy in selected patients. Immunotherapeutic investigations further include LMP1/2-specific cytotoxic T lymphocytes and CAR-T cells, which have shown preliminary anti-tumor activity. Collectively, these targets and approaches provide new avenues for precision therapy of EBV^+^ HL.

## 10 Discussion

Epstein–Barr virus (EBV), the first human oncogenic virus ever identified, has been the subject of intensive mechanistic investigation for more than half a century. Here we systematically delineate the diverse spectrum of Epstein-Barr virus-associated malignancies, spanning epithelial and lymphoid origins. Despite their disparate tissue origins, clinical presentations, and distinct epidemiological patterns, a central theme emerges: their shared viral etiology implies the existence of convergent oncogenic principles. By transcending the boundaries of individual tumor types, we can identify a commonalities in these cancers, paving the way for pan-tumoral, EBV-directed therapeutic strategies.

A cornerstone of EBV’s oncogenic strategy is its ability to adopt specific latency programs tailored to the host cell context, yet these programs converge on a limited set of critical cellular pathways. From primary infection through lifelong persistence, EBV displays distinct phenotypes in B cells versus epithelial cells, yet converges on a common transformation route centred on immune escape, survival advantage and clonal expansion. The type II latency program, characteristic of NPC, EBVaGC, cHL, NKTCL, and LEC, consistently utilizes LMP1 and LMP2A to constitutively activate the NF-κB and PI3K/AKT signaling axes, driving proliferation and survival. This mimics chronic antigen receptor stimulation, driving proliferation and survival. Even in tumors with more restricted latency, such as the EBNA1-only Latency I in BL and EBVaICC, the virus provides crucial survival signals by blocking apoptosis and driving cell-cycle progression. In BL, EBNA1 and viral non-coding RNAs counteract the profound pro-apoptotic signals induced by the defining MYC translocations. Across all malignancies, EBV orchestrates widespread epigenetic silencing of tumor suppressor genes, as starkly demonstrated by the CpG island methylator phenotype (CIMP) in EBVaGC. Furthermore, the boundary between latency and lytic replication is blurred, with lytic proteins like BHRF1 and BALF1 contributing to survival and genomic instability even in predominantly latent tumors, underscoring a fluid viral life cycle that fuels oncogenesis.

A fascinating and clinically critical commonality among EBV-associated tumors is their “immune-hot” TME, characterized by dense lymphocytic infiltration. This is most evident in EBVaGC, LEC, and NPC, where the stroma is rich in T cells, B cells, and myeloid cells. However, this robust immune infiltration is paradoxically coupled with profound functional suppression. The virus orchestrates a multi-layered immune evasion strategy: 1. Impaired Antigen Presentation: A universal strategy involves downregulation of the antigen presentation machinery. This is achieved through EBNA1’s Gly-Ala repeat domain inhibiting its own processing, viral proteins like BNLF2a interfering with TAP function, and LMP1/LMP2-driven downregulation of HLA molecules. 2. Exploitation of Immune Checkpoints: The consistent upregulation of the PD-1/PD-L1 axis across NPC, EBVaGC, cHL, DLBCL, and NKTCL is a hallmark of EBV-driven immune evasion. LMP1 is a key driver of PD-L1 expression, and clinical evidence from EBVaGC and EBVaICC confirms that this renders tumors susceptible to PD-1/PD-L1 blockade. 3. Recruitment and Polarization of Suppressive Cells: EBV reshapes the cellular composition of the TME towards an immunosuppressive state. This includes the enrichment of regulatory T cells (Tregs), often characterized by specific markers like LAG3 or TNFRSF4, and the polarization of tumor-associated macrophages (TAMs) towards an M2 phenotype, as seen in DLBCL and BL. The role of B cells is complex and stage-dependent, acting as antitumor effectors early on but potentially adopting immunosuppressive (Breg) functions in advanced disease.

Genetic Susceptibility and Immune Control is one of the major concern regarding EBV associated malignancies. The geographic and ethnic disparities in EBV-associated cancers are heavily influenced by host genetics, particularly the HLA system. Specific HLA alleles confer risk (e.g., HLA-A*02:07 and HLA-B*46:01 in NPC; specific HLA-DP/DR haplotypes in NKTCL) or protection (e.g., HLA-A*11:01 in NPC; HLA-A*02:01 in NKTCL and cHL), primarily by modulating the efficiency of EBV antigen presentation to T cells. Furthermore, the interplay between high-risk EBV strains and specific HLA alleles can further amplify disease susceptibility through epitope variation. This genetic landscape creates a population-level “immune bottleneck, “ where individuals with less efficient viral control are predisposed to EBV-driven oncogenesis.

**Figure 9:**
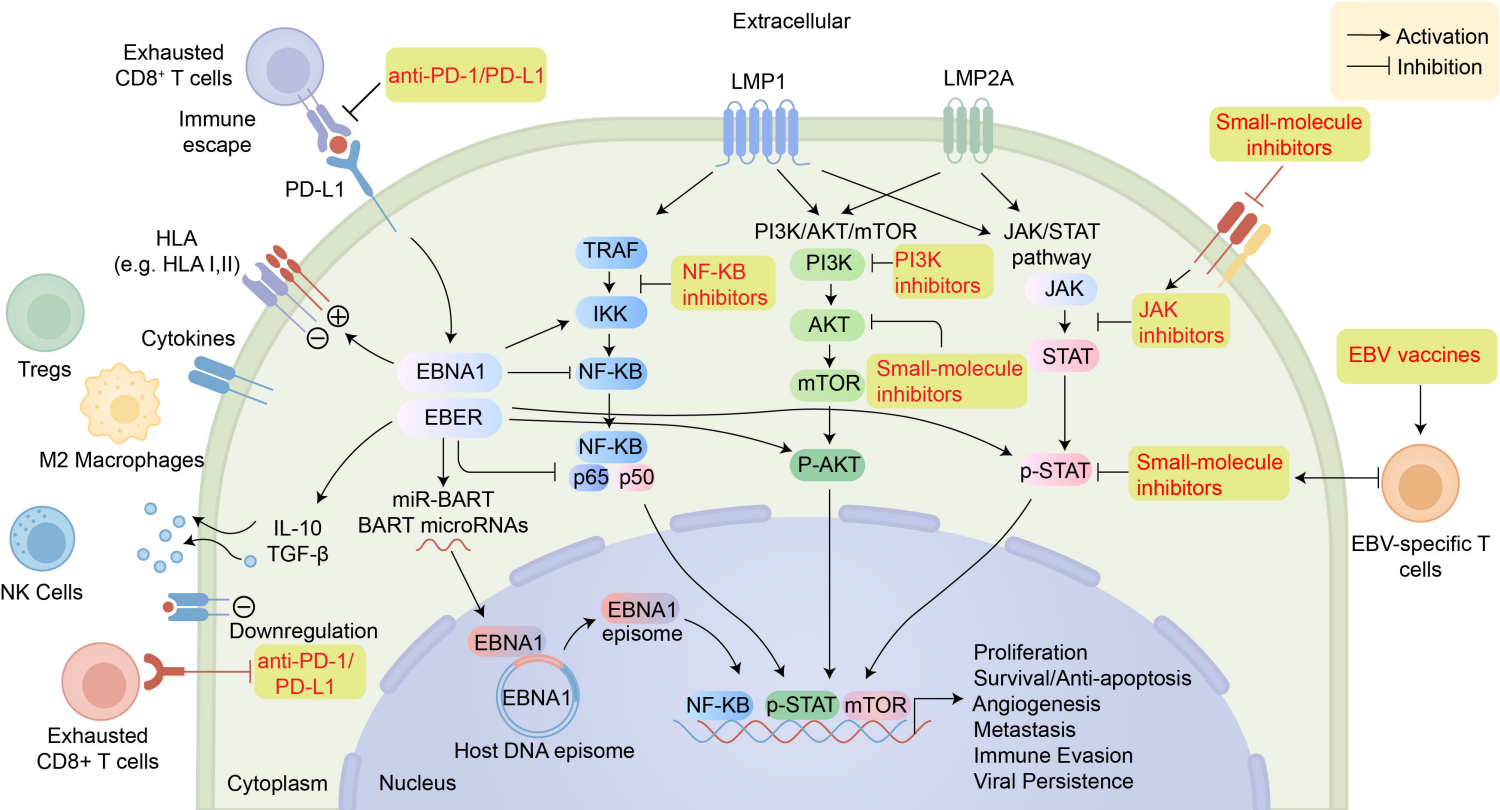
Integrated landscape of shared oncogenic signaling and rational pan-tumoral therapeutic interventions in EBV-associated malignancies. EBV-encoded oncoproteins (LMP1, LMP2A, EBNA1) and non−coding RNAs (EBERs, BART miRNAs) drive sustained activation of the NF−κB, PI3K/AKT/mTOR, and JAK/STAT signaling pathways, promoting cell proliferation, survival, angiogenesis, metastasis, and immune evasion. Concurrently, EBV induces HLA downregulation, PD−L1 upregulation, and the accumulation of immunosuppressive cells (Tregs, M2 macrophages, exhausted CD8^+^ T cells), establishing an immune−suppressive tumor microenvironment. Corresponding pan−tumor therapeutic strategies are highlighted, including immune checkpoint inhibitors (anti−PD−1/PD−L1), EBV−targeted vaccines, EBV−specific T−cell therapies, and small−molecule inhibitors against key signaling nodes. This integrated diagram summarizes the convergent pathogenic features and actionable therapeutic vulnerabilities across diverse EBV−driven cancers.

The shared biology of EBV-associated malignancies not only reveals common oncogenic principles but also establishes a rational foundation for developing pan-tumoral therapeutic strategies. Moving away from the pursuit of isolated modalities, contemporary approaches are increasingly designed to concurrently target interconnected vulnerabilities. These span the intrinsic viral life cycle, the host signaling networks hijacked by EBV, and the profoundly immunosuppressive microenvironment orchestrated by the virus. Herein, we delineate a multi-layered framework that translates these biological convergences into a cohesive therapeutic roadmap, thereby transcending traditional histology-specific boundaries to embrace a unified, virus-centric oncological paradigm (**Figure 9**). To illustrate the translational landscape of this framework, **Table 2** systematically summarizes key clinical trials encompassing these diverse therapeutic layers.

**Table 2:** The completed and recruiting clinical trials of EBV-associated tumor.

Tumor type	Trial ID/Name	Interventions	Patients(N)	Phase	Status
EBV^+^NPC	NCT02065362	DNR.NPC-specific T cells + cyclophosphamide + fludarabine	40	PHASE 1	ACTIVE_NOT_RECRUITING
Locally advanced NPC with detectable EBV DNA	NCT05772208	Camrelizumab Nimotuzumab	15	PHASE 3	RECRUITING
Locally advanced NPC with detectable EBV DNA	NCT05628922	Toripalimab	198	PHASE 2	RECRUITING
EBV^+^Lymphoma EBV^+^Malignancies	NCT02287311	MABEL CTLs Cyclophosphamide Fludarabine	40	PHASE 1	RECRUITING
R/R EBV^+^NPC	NCT05587543	EBV CAR-T/TCR-Tcell	24	EARLY_PHASE 1	RECRUITING
EpCAM^+^patients with Advanced Solid Tumors	NCT02915445	EpCAM CAR-T cells	30	PHASE 1	RECRUITING
EBV^+^GC	NCT03257163	Pembrolizumab, Capecitabine, and Radiation Therapy	40	RECRUITING	RECRUITING
Advanced EBV^+^Solid Tumors	NCT05166577	Nanatinostat Plus Valganciclovir	130	PHASE 1 PHASE 2	RECRUITING
EBV^−^Refractory Malignant Tumors	NCT05714748	mRNA Vaccine	9	PHASE 1	RECRUITING
EBV^+^GC/NKTCL	NCT03789617	EBViNT Cell	72	PHASE 1	RECRUITING
EBV^+^GC	NCT03257163	Pembrolizumab, Capecitabine, and Radiation Therapy	40	PHASE 2	RECRUITING
EBV^+^Advanced GC	NCT05970627	Poripalimab Oxaliplatin S1	30	PHASE 2	NOT YET RECRUITING
EBV^+^Iymphoma	NCT04664179	C7R-EBV T cells	44	PHASE 1	RECRUITING
CD30^+^lymphoma	NCT06176690	C7R.CD30.CAR-EBVST cells	90	PHASE 1	RECRUITING
NKTCL	NCT06069830	mid-term PET and EBV DNA-directed therapy	89	PHASE 2	RECRUITING
EBV^+^lymphoma EBV^+^malignancies	NCT02287311	MABEL CTLs Cyclophosphamide Fludarabine	42	PHASE 1	RECRUITING
NKTCL	NCT06255795	Chidamide Anti-PD1 antibody Pegaspargase DDGP	142	PHASE 3	NOT YET RECRUITING
EBV^+^Lymphoma disorders	NCT03258567	Nivolumab	40	PHASE 2	RECRUITING
NKTCL		Linperlisib Camrelizumab Pegaspargase	NCT06376721	PHASE 1 PHASE 2	RECRUITING
CD30^+^lymphoma	NCT04288726	CD30 CAR-EBVST cells	18	PHASE 1	RECRUITING
EBV^+^R/R lymphoma	NCT05011058	Nanatinostat Valganciclovir	140	PHASE 2	RECRUITING
T-cell Lymphoma	NCT06224049	hNeo-T Cyclophosphamide Fludarabine	6	EARLY PHASE 1	RECRUITING

### 10.1 Targeting the viral life cycle

The restricted expression of latent proteins (LMP1, LMP2, EBNA1) across latency II/III tumors offers direct and highly specific targets. Adoptive transfer of EBV-specific cytotoxic T lymphocytes (EBV-CTLs) has achieved durable remissions in LEC and post-transplant lymphoproliferative disorders. TCR-T cells targeting LMP1/2 and EBNA1, and CAR-T cells against viral or virus-induced surface markers (e.g., LMP2-CAR), represent the next frontier, though their efficacy in solid tumors remains under investigation (215, 292). Alternatively, targeting the latent-to-lytic switch represents a potent “reactivation-elimination” strategy. Epigenetic modulators (e.g., HDAC inhibitors or demethylating agents) can pharmacologically force lytic reactivation, thereby sensitizing latently infected cells — such as NPC, LEC, and cHL — to antiviral prodrugs (e.g., ganciclovir) and amplifying immune recognition (78). Furthermore, neutralizing EBV-encoded non-coding RNAs — using TLR3 antagonists against EBERs or antisense oligonucleotides against BART miRNAs — demonstrates robust preclinical efficacy in dismantling viral-driven survival networks (45, 298).

### 10.2 Intercepting virus-hijacked host pathways

To counter the constitutive activation of host signaling pathways (NF-κB, PI3K/AKT, JAK/STAT) driven by viral latent proteins, combination regimens incorporating small molecule inhibitors against these pathways may synergize with immune-based therapies. Robust preclinical evidence across diverse EBV-associated malignancies demonstrates that PI3K/mTOR or JAK2 blockade can reverse LMP-driven immune evasion and enhance T-cell effector function. Small molecule inhibitors targeting these pathways—such as PI3K/mTOR inhibitors, bortezomib, and JAK inhibitors—hold therapeutic potential, particularly in tumors with concurrent host mutations (e.g., PIK3CA in EBVaGC, IDH1/FGFR2 in EBVaICC). Such combinations represent a logical extension of “virus-host co-targeting, “ creating a powerful bifocal approach that simultaneously targets viral drivers and host dependencies.

### 10.3 Immune reinvigoration in the tumor microenvironment

The immunosuppressive TME, which is characterized by PD-L1 upregulation, regulatory T−cell infiltration, and M2 macrophage polarization, provides a strong biological rationale for developing combinatorial immunomodulatory strategies. Immune checkpoint blockade, particularly targeting PD-1/PD-L1, has demonstrated efficacy across multiple EBV-associated cancers, with pembrolizumab and nivolumab showing clinical benefit in NPC (KEYNOTE-028), EBVaGC, and cHL (308, 309). Plasma EBV DNA load has become a real-time prognostic biomarker guiding risk stratification and response monitoring. However, response rates remain variable, highlighting the need for biomarker-driven strategies and rational combinations. Integrating PD-1/PD-L1 inhibitors with next-generation checkpoint inhibitors including LAG-3, TIGIT, and TIM-3 holds promise for reversing terminal CD8^+^ T-cell exhaustion.

### 10.4 Priming and expanding EBV-specific immunity with therapeutic vaccines

The “immune-hot” but dysfunctional TME provides a strong rationale for incorporating therapeutic vaccines as a priming strategy. Unlike passive immunotherapies, active vaccination can expand and diversify EBV-specific T-cell clones, potentially converting the exhausted infiltrate into a sustained effector pool. Recent advances have focused on viral antigens expressed during EBV latency, particularly LMP2, which is stably expressed across type II latency tumors including NPC, EBVaGC, and cHL, and EBNA1, which is universally present across all latency types. A lipid-encapsulated LMP2 mRNA vaccine (LPX-mLMP2) has demonstrated robust CD8^+^ T-cell priming and tumor growth inhibition in preclinical models (108). Building upon this concept, a multi-antigen mRNA vaccine incorporating truncated forms of EBNA1, EBNA3A, and LMP2A has been evaluated in preclinical mouse models, where it elicited broader cellular and humoral immune responses compared to single-antigen formulations, with the truncated antigen designs demonstrating superior immunogenicity (109). Notably, the AI-designed EBV mRNA vaccine WGc-043 (NCT05714748) has entered clinical testing in recurrent/metastatic NPC, achieving a disease control rate of 66.67% with acceptable safety. Recently, a “universal anti-EBV cellular vaccine” technology has also been successfully applied in the clinic. As a therapeutic vaccine, it can elicit robust EBV-specific T-cell responses and reducing viral DNA load. Its “universal” design and preliminary clinical data (NCT05707910), which demonstrate a disease control rate of 83.3%, provide a new strategic option that expands the therapeutic arsenal for EBV−associated diseases. Looking forward, the strategic positioning of therapeutic vaccines is likely to be in combination with immune checkpoint inhibitors: vaccines can expand the T-cell repertoire, while checkpoint blockade prevents exhaustion of those newly activated clones. Such “prime-and-checkpoint” regimens are currently being explored in EBV-positive gastric cancer and NPC, and their success will depend on careful sequencing, optimal antigen selection, and integration with other immunomodulatory agents.

## 11 Conclusion and future perspectives

In conclusion, the remarkable oncogenic versatility of EBV is counterbalanced by a set of unifying principles. The future of therapy lies in moving beyond a histology-specific paradigm towards a molecularly guided, virus-centric approach. Vaccination is viewed as the ultimate tool to terminate the EBV-related cancer wave, though its development is constrained by undefined correlates of protection and the difficulty of eradicating the latent reservoir. Should a sterilizing vaccine ultimately emerge, it would not only establish immune protection but also definitively test the necessity and sufficiency of EBV in carcinogenesis. Until then, the most promising path forward involves rational combinations of immune checkpoint inhibitors, next-generation cellular therapies, mRNA vaccines, and pathway-specific small molecules. By targeting the common viral engine that drives these diverse cancers through integrated “virus-targeted + immune-modulatory” regimens, we can develop more effective and precise pan-tumoral strategies to improve outcomes for patients worldwide.
